# Electroacupuncture inhibits pyroptosis to improve motor function in an MPTP-induced mouse model of Parkinson’s disease

**DOI:** 10.3389/fnagi.2026.1968826

**Published:** 2026-09-30

**Authors:** Yu-Chen Ying, Yi-Yue Dong, Guang-Yan Chen, Xi Wang, Qian Fan, Xi-Chen Wu, Yue-Lai Chen

**Affiliations:** 1 Longhua Hospital Shanghai University of Traditional Chinese Medicine, Shanghai, China; 2 Gansu Provincial People’s Hospital, Jiangsu University, Lanzhou, Gansu, China; 3 Ningbo Hospital of Integrated Traditional Chinese and Western Medicine, Ningbo, Zhejiang, China

**Keywords:** electroacupuncture, MCC950, Nigericin, NLRP3, Parkinson’s disease

## Abstract

**Objectives:**

To investigate whether electroacupuncture (EA) ameliorates motor dysfunction and protects nigrostriatal dopaminergic neurons in Parkinson’s disease (PD) mice by inhibiting NLRP3 inflammasome-mediated pyroptosis.

**Methods:**

Male C57BL/6 mice were randomly assigned before model induction to seven groups: saline-treated control, MPTP model, MPTP plus EA, MPTP plus Nigericin, MPTP plus EA and Nigericin, MPTP plus MCC950, and MPTP plus EA and MCC950 groups. Mice in the control group received equal-volume saline, whereas mice in the other six groups were administered 1-methyl-4-phenyl-1,2,3,6-tetrahydropyridine (MPTP; 30 mg/kg, intraperitoneally) once daily for five consecutive days. Following model induction, EA was applied at Baihui (GV20) and Dazhui (GV14) at 100 Hz for 20 min per session over 14 days. Nigericin and MCC950 were used as an NLRP3 inflammasome activator and inhibitor, respectively. Motor function was assessed using the open-field and pole tests. Dopaminergic neuronal integrity, neuronal injury, microglial activation, and the cellular localization of NLRP3 were evaluated using immunohistochemistry, hematoxylin–eosin staining, and immunofluorescence. The expression levels of tyrosine hydroxylase, NLRP3, ASC, Caspase-1, and the N-terminal fragment of gasdermin D (GSDMD-N) were determined using immunofluorescence and western blotting.

**Results:**

EA alleviated abnormal open-field hyperlocomotion and prolonged pole-descent latency in MPTP-treated mice, preserved tyrosine hydroxylase-positive dopaminergic neurons in the substantia nigra, restored striatal tyrosine hydroxylase expression, and reduced neuronal injury and microglial activation. NLRP3 was predominantly colocalized with tyrosine hydroxylase-positive dopaminergic neurons, whereas limited colocalization was observed with IBA-1-positive microglia. Electroacupuncture significantly suppressed the MPTP-induced upregulation of NLRP3, ASC, Caspase-1, and GSDMD-N. Nigericin exacerbated motor abnormalities, nigrostriatal injury, and pyroptosis-related signaling and largely abolished the protective effects of EA. Conversely, MCC950 produced protective effects comparable to those of EA, whereas their combined administration provided no significant additional benefit.

**Conclusion:**

EA improves motor dysfunction and protects the nigrostriatal dopaminergic system in MPTP-induced PD mice, at least partly by suppressing NLRP3 inflammasome-mediated pyroptosis in dopaminergic neurons. These findings identify the NLRP3 inflammasome as an important mechanistic target underlying the neuroprotective effects of electroacupuncture in experimental PD.

## Introduction

Parkinson’s disease (PD) is the second most common neurodegenerative disease after Alzheimer’s disease (AD), with an estimated global prevalence of 7–10 million patients (Granado et al., 2013). A recent systematic review on PD incidence reported overall rates of 61.21 and 37.55 per 100,000 population for men and women over 40 years of age, respectively (Hirsch et al., 2016). The incidence in both sexes increases with age (Hirsch et al., 2016). PD is characterized by a substantial (50%–70%) loss of dopaminergic neurons in the substantia nigra (SN) pars compacta that project to the striatum (Gandhi and Wood, 2005). The primary symptoms of PD include a range of motor and non-motor features, such as resting tremor, bradykinesia, rigidity, and loss of postural reflexes (Jankovic, 2008). While genetic and environmental risk factors are associated with PD etiology, the primary cause remains unclear (Bernhardt and Schulze-Hentrich, 2025; Stoessl, 1999). PD is one of the most severe chronic neurological disorders, leading to a significant decline in patients’ quality of life (Ward, 2000). The main types of medications available for treating PD symptoms include: levodopa, dopamine agonists, MAO-B inhibitors, COMT inhibitors, amantadine, and anticholinergic agents (Fahn et al., 2004; Goldenberg, 2008). Current treatments for Parkinson’s disease aim to reduce motor impairment by maintaining normal dopamine (DA) levels, inhibiting the degradation of endogenous DA, and supplementing with the DA precursor levodopa or DA agonists. Initially, pharmacotherapy may significantly improve PD symptoms; however, the benefits often diminish or become less stable over time. Deep brain stimulation surgery has been demonstrated to improve motor symptoms and quality of life in patients with advanced PD (Deuschl et al., 2006). Nevertheless, these treatments have shown only limited effectiveness in improving clinical symptoms and are associated with adverse side effects, such as motor fluctuations and dyskinesias (Prasad and Hung, 2021). There are currently no disease-modifying therapies for PD patients, making the search for safer and more effective treatments an urgent and critical challenge.

Current research indicates that sustained and uncontrolled activation of the NLRP3 inflammasome plays a central role in the pathophysiology of neuroinflammation in age-related neurodegenerative diseases such as PD and AD (Broz and Dixit, 2016). Inflammasomes are components of the immune response initiation to various harmful stimuli and are multiprotein complexes assembled in response to cytosolic danger or damage-associated signals (Swanson et al., 2019). The NOD-, LRR- and pyrin domain-containing protein 3 (NLRP3) is a cytosolic sensor that can initiate inflammasome activation in response to a range of damage signals. The NLRP3 inflammasome consists of a sensor protein (NLRP3), an adaptor component (ASC or PYCARD), and an effector (Caspase-1) (Possemato et al., 2023). Upon assembly, the inflammasome cleaves cytokines and other targets, ultimately leading to cell lysis and the release of active cytokines. Active caspase-1 cleaves gasdermin D (GSDMD), forming gasdermin pores in the cell membrane and resulting in a pro-inflammatory cell death known as pyroptosis (Possemato et al., 2023).

Acupuncture, a therapeutic modality widely utilized in Traditional Chinese Medicine, has a long history in the management of PD (World Health Organization, 2002). In China, acupuncture is a common treatment option for many clinicians and PD patients (Lin and Chen, 2008). In clinical practice, it is a commonly adopted intervention for PD patients in China (Lin and Chen, 2008), with reported benefits including significant improvements in total Unified Parkinson’s Disease Rating Scale (UPDRS) scores (Wang et al., 2008). Notably, 70%–80% of patients have reported subjective symptom relief, enhanced motor function, as well as improvements in sleep quality and mood following acupuncture therapy (Li et al., 2025; Rajendran et al., 2001). Since the 1970s, electroacupuncture (EA) has gained increasing popularity and is often the preferred acupuncture modality for researchers, as it facilitates standardization of the stimulation type and quantity for each subject compared to traditional manual acupuncture. Recent findings indicate that PD patients receiving EA treatment experienced improvements in rigidity, balance, and sleep quality (Tan et al., 2006). Animal studies have also confirmed that EA at Baihui (GV20) and Dazhui (GV14) acupoints reduces rotational behavior and enhances the survival of dopaminergic neurons in PD rats (Liang et al., 2002, 2003). Similarly, in MPTP-induced PD mice, EA stimulation has been shown to ameliorate behavioral deficits, protect dopaminergic neurons, and restore striatal dopamine levels (Kang et al., 2007). Despite accumulating evidence of its efficacy, the precise mechanisms underlying EA’s neuroprotective effects remain incompletely understood. While it has been suggested that EA may inhibit the expression of NLRP3 in the SN and striatum of 6-Hydroxydopamine-induced rats, the involvement of this inflammasome pathway in EA-mediated neuroprotection has yet to be elucidated (Liu et al., 2022). Therefore, the present study was designed to investigate the therapeutic effects of EA on PD and to determine whether inhibition of the NLRP3 inflammasome-mediated pyroptosis contributes to the amelioration of motor deficits in MPTP mice.

## Materials and methods

### Animals

Eight-week-old male C57BL/6 mice (weighing 23–25 g) were purchased from Shanghai Xipur-Biak Co., Ltd. All animals were maintained in a specific pathogen-free (SPF) facility under controlled environmental conditions (temperature: 21 °C–23 °C; humidity: 50%–60%) with free access to standard food and water. The study protocol was approved by the Animal Ethics Committee of Longhua Hospital (Approval No. LHERWA-25038), and all procedures conformed to international standards for the care and use of laboratory animals.

### MPTP-induced PD mouse model

After a 7-day acclimatization period in a controlled environment, the mice were randomly assigned to seven experimental groups. The control group received daily intraperitoneal (i.p.) injections of sterile saline (volume-matched to the MPTP-treated groups). Parkinson’s disease models were established by administering MPTP (30 mg/kg, i.p.) for five consecutive days. EA was performed 1 h after MPTP injection on days 1–5, and EA was also performed on days 8–12. To investigate the involvement were of the NLRP3 inflammasome, two additional treatment regimens implemented: the MPTP+Nigericin group received i.p. injections of Nigericin (2 mg/kg, an NLRP3 agonist), while the EA+Nigericin group received the same Nigericin treatment alongside EA intervention. Conversely, the MPTP+MCC950 group received i.p. injections of MCC950 (10 mg/kg, a specific NLRP3 inhibitor), and the EA+MCC950 group received MCC950 in combination with EA treatment. All pharmacological agents, including MPTP (S4732, Selleck, USA), Nigericin (S6653, Selleck, USA), and MCC950 (S8930, Selleck, USA), were obtained from Selleck Chemicals. EA stimulation was delivered using an SDZ-V electroacupuncture device (Huatuo, China). Sterile disposable acupuncture needles [Huatuo, Suzhou Medical Appliance Factory Co., Ltd., Suzhou, China; 0.30 mm in diameter × 13 mm in length (0.5 inch)] were inserted at GV20 and GV14 to a depth of approximately 2–3 mm. The positive electrode was connected to the needle at GV20, and the negative electrode was connected to the needle at GV14. Stimulation was delivered in continuous-wave mode at a frequency of 100 Hz for 20 min per session. The current intensity was individually adjusted within the range of 0.2–1.0 mA until a slight bilateral twitch of the ears was observed, which was used as the operational criterion for adequate stimulation. During EA stimulation, the mice were maintained under isoflurane anesthesia. Treatments were administered once daily, 5 days per week, for a total of 14 consecutive days. Nigericin (2 mg/kg) and MCC950 (10 mg/kg) were administered via intraperitoneal injection once every 2 days, three times per week, for a total of six injections. All injections were performed 1 h prior to each EA session. On day 15 following the initial MPTP injection, all animals were deeply anesthetized using a gas anesthesia system (2% isoflurane; R500IP, RWD) and subsequently perfused transcardially with ice-cold phosphate-buffered saline. Whole brain tissues were promptly harvested and processed for further histological and biochemical analyses.

### Pole test

Motor coordination and bradykinesia were evaluated using a pole descent test. In this assay, each mouse was positioned facing upward at the top of a rough-surfaced vertical pole (55 cm in height, 1 cm in diameter) wrapped with medical gauze to ensure adequate grip. The apparatus was placed adjacent to the animal’s home cage to encourage natural downward movement. The time taken for the mouse to descend from the starting point to the bottom of the pole was recorded as the primary endpoint, reflecting total descent latency.

### Open field test

Spontaneous locomotor activity and anxiety-like behavior were assessed using an open field test. Each mouse was gently placed in the periphery of a novel arena (50 × 50 × 40 cm) under dim lighting conditions and allowed to explore freely for 10 min. Locomotor trajectories were recorded via an overhead camera and analyzed using VisuTrack software (XinRuan, China). The following parameters were quantified: (1) time spent in the central zone (10 × 10 cm square); (2) total distance traveled; and (3) the number of entries into both peripheral (within 15 cm of the walls) and central regions. Between each trial, the apparatus was thoroughly cleaned with 75% ethanol and allowed to air-dry to eliminate olfactory cues. Locomotor activity was evaluated based on total distance moved, average speed, and total ambulatory time.

### Immunohistochemistry (IHC) staining

Following euthanasia under deep anesthesia, mice were perfused transcardially with 0.9% saline, followed by ice-cold 4% paraformaldehyde (PFA). Extracted brains were immersed in fresh 4% PFA at 4 °C for 24 h for post-fixation and subsequently embedded in paraffin. Serial coronal sections (5 μm thick) were prepared, dewaxed, and subjected to heat-induced antigen retrieval. For immunohistochemical staining, sections were incubated with rabbit anti-tyrosine hydroxylase (TH) (AF6113, Affinity Biosciences, China) for 60 min at room temperature (RT). After washing, the sections were exposed to HRP-conjugated goat anti-rabbit IgG (S0001, Affinity Biosciences, China) for 30 min, followed by visualization with DAB chromogen and counterstaining with hematoxylin. Images were acquired using an Olympus IX73 inverted microscope.

### Immunofluorescence (IF) staining

Brain paraffin sections were dewaxed in xylene and rehydrated through a descending ethanol series. Antigen retrieval was achieved by microwave heating in EDTA buffer (pH 8.0). After blocking with normal serum for 1 h to reduce non-specific binding, the sections were incubated overnight at 4 °C with primary antibodies targeting key proteins involved in pyroptosis, including anti-NLRP3 (BF8029, Affinity Biosciences, China), anti-IBA-1 (DF6442, Affinity Biosciences, China), anti-Caspase-1 (AF5418, Affinity Biosciences, China), anti-ASC (DF6304, Affinity Biosciences, China), anti-GSDMD-N (DF13758, Affinity Biosciences, China), and β-actin (AF7018, Affinity Biosciences, China). Following three rinses in PBS, the sections were exposed to appropriate fluorophore-conjugated secondary antibodies for 1 h at room temperature in the dark. Nuclei were counterstained with DAPI where applicable. Fluorescent images were captured using an Olympus BX61 epifluorescence microscope equipped with filter sets specific for each fluorochrome.

### Western blotting (WB)

Brain tissues were lysed using RIPA buffer (P0013B, Beyotime, China) supplemented with phenylmethylsulfonyl fluoride. Following centrifugation at 12,000 × *g* for 10 min at 4 °C, the supernatant was collected and protein concentrations were determined with a BCA protein assay kit (23227, Pierce, Waltham, USA). Equal amounts of protein were resolved by 10% SDS-PAGE and electrotransferred onto PVDF membranes. Membranes were blocked with 5% non-fat milk in TBST for 1 h at room temperature, then incubated overnight at 4 °C with primary antibodies recognizing NLRP3 (BF8029, Affinity Biosciences, China), IBA-1 (DF6442, Affinity Biosciences, China), Caspase-1 (AF5418, Affinity Biosciences, China), ASC (DF6304, Affinity Biosciences, China), GSDMD-N (Affinity Biosciences, China), TH (1:1000, AF6113, Affinity Biosciences, China), and β-actin (AF7018, Beyotime, China). After washing, the blots were incubated with HRP-conjugated secondary antibodies for 1 h at room temperature. Immunoreactive bands were visualized using enhanced chemiluminescence reagents (SQ201, Yamei, China) and imaged with a chemiluminescence detection system.

### Hematoxylin and eosin (HE) staining

Brain sections were deparaffinized in xylene and rehydrated through a graded ethanol series. The sections were stained with Harris hematoxylin solution for 5 min to visualize nuclei, followed by differentiation in 1% acid alcohol and bluing in tap water. Subsequently, the sections were counterstained with 0.5% eosin Y solution for 2 min to stain the cytoplasm. After dehydration through graded ethanol and clearing in xylene, the sections were mounted with neutral balsam. Images were captured using a light microscope (Olympus, Japan) to evaluate neuronal morphology and pathological changes.

### Statistical analysis

All data were presented as mean ± standard deviation (SD). Prior to analysis, normality and homogeneity of variance were assessed using the Shapiro-Wilk test and Brown-Forsythe test, respectively. As the data met the assumptions of parametric testing, statistical comparisons among multiple groups were conducted using one-way analysis of variance (ANOVA), followed by Tukey’s *post-hoc test* for pairwise comparisons. All statistical analyses were performed using GraphPad Prism 9.0 (GraphPad Inc., USA), and a *p*-value of less than 0.05 was considered statistically significant.

## Results

### EA improved motor function in MPTP mice

In the open field test, mice in the MPTP group exhibited significant alterations in locomotor activity compared to the control group. Specifically, they showed (1) a decrease in the number of central zone crossings (Figures 1A,B); (2) a reduction in time spent in the central zone, accompanied by a corresponding increase in time spent in the peripheral zone (Figures 1A,B); and (3) a significant increase in both average movement speed and total distance traveled (Figures 1C,D). EA intervention effectively reversed these behavioral abnormalities, as evidenced by prolonged time spent in the central zone (Figures 1A,B), reduced time in the peripheral zone (Figures 1A,B), and decreased average movement speed and total distance traveled (Figures 1C,D). Furthermore, the pole test revealed that mice in the MPTP group took significantly longer to descend from the top to the bottom of the pole compared to the control group, whereas EA treatment markedly shortened this descent time (Figure 1E).

**FIGURE 1:**
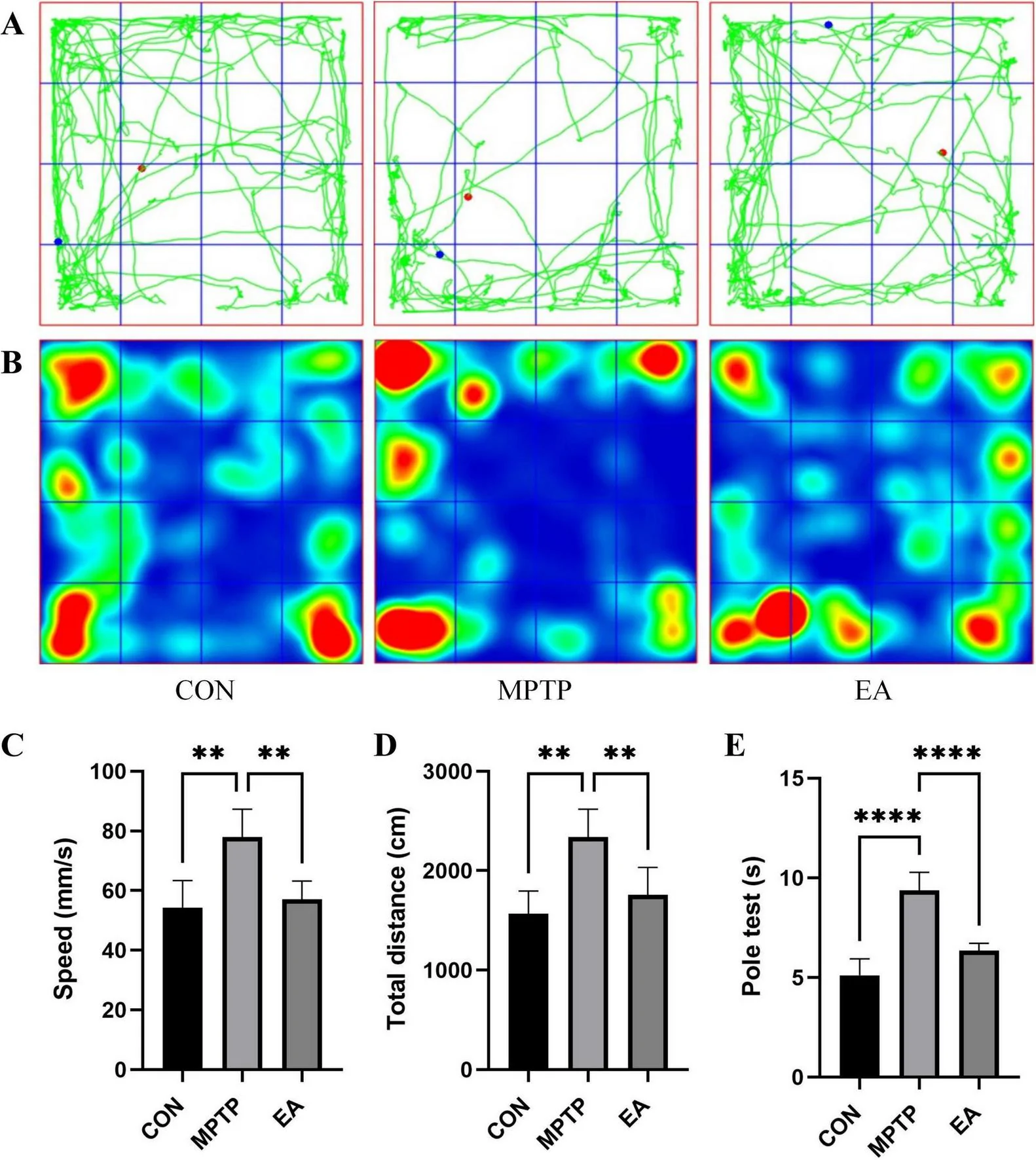
Effects of electroacupuncture (EA) on motor functions in MPTP-induced Parkinson’s disease (PD) mice. **(A)** Representative trajectories of mice in the open field. **(B)** Cumulative heatmap of time spent in the open field. **(C)** Average moving speed of the three groups of mice. **(D)** Total travel distance of the three groups of mice in the open field. **(E)** Time spent in the pole test. Data are means ± SD. *n* = 5, **P < 0.01, ****P < 0.0001.

### Neuroprotective effects of EA on the nigrostriatal pathway in MPTP mice

Immunohistochemistry revealed that, compared to the control group, the expression levels of TH in the SN and striatum were markedly reduced in the MPTP group (Figures 2A,B and Supplementary Tables 1, 2). Notably, this reduction was restored following EA intervention in the corresponding brain regions (Figures 2A,B and Supplementary Tables 1, 2). Consistent with this, HE staining indicated that the MPTP group exhibited neuronal necrosis, whereas EA treatment effectively ameliorated these pathological alterations (Figure 2C and Supplementary Table 3). Furthermore, IF analysis demonstrated that microglia were activated in the model group, while this activation was suppressed by EA treatment (Figure 2D and Supplementary Table 4). Quantitative analysis confirmed the changes observed in the representative immunohistochemical images, and the complete numerical results and statistical comparisons are provided in Supplementary Tables 1–4.

**FIGURE 2:**
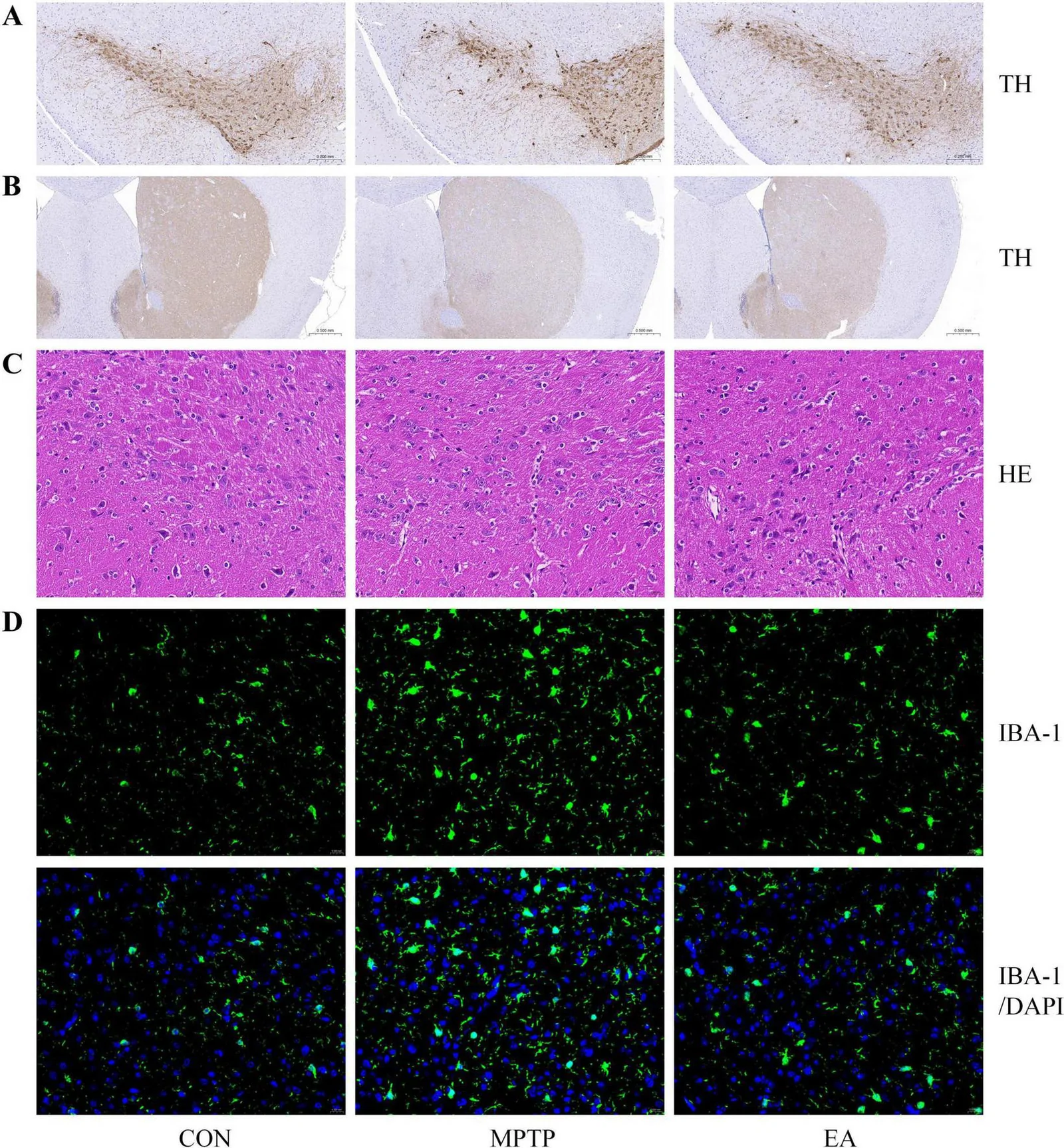
Electroacupuncture (EA) alleviates MPTP-induced dopaminergic neuron loss and neuroinflammation in mice. **(A)** Immunohistochemistry (IHC) staining for tyrosine hydroxylase (TH) in the substantia nigra (SN). **(B)** IHC staining for TH in the striatum. **(C)** Hematoxylin and eosin (HE) staining in the SN. **(D)** Immunofluorescence (IF) staining for IBA-1 in the SN. *n* = 5.

### EA inhibits NLRP3 expression in dopaminergic neurons of the SN in MPTP mice

NOD-, LRR- and pyrin domain-containing protein 3 is expressed in multiple cell types within the brain; however, current research has primarily focused on microglia and dopaminergic neurons (Bloem et al., 2021; Liu et al., 2021). To investigate its localization with respect to dopaminergic neurons and microglia, IF staining was performed in this study. The results showed that, compared with the control group, NLRP3 expression was upregulated in the SN of MPTP mice (Figures 3A,B). Following EA intervention, NLRP3 expression in this brain region was reduced (Figures 3A,B). Co-localization analysis further revealed weak overlapping signals between IBA-1 and NLRP3 (Figure 3C), whereas a strong co-localization signal was observed between TH and NLRP3 (Figure 3D).

**FIGURE 3:**
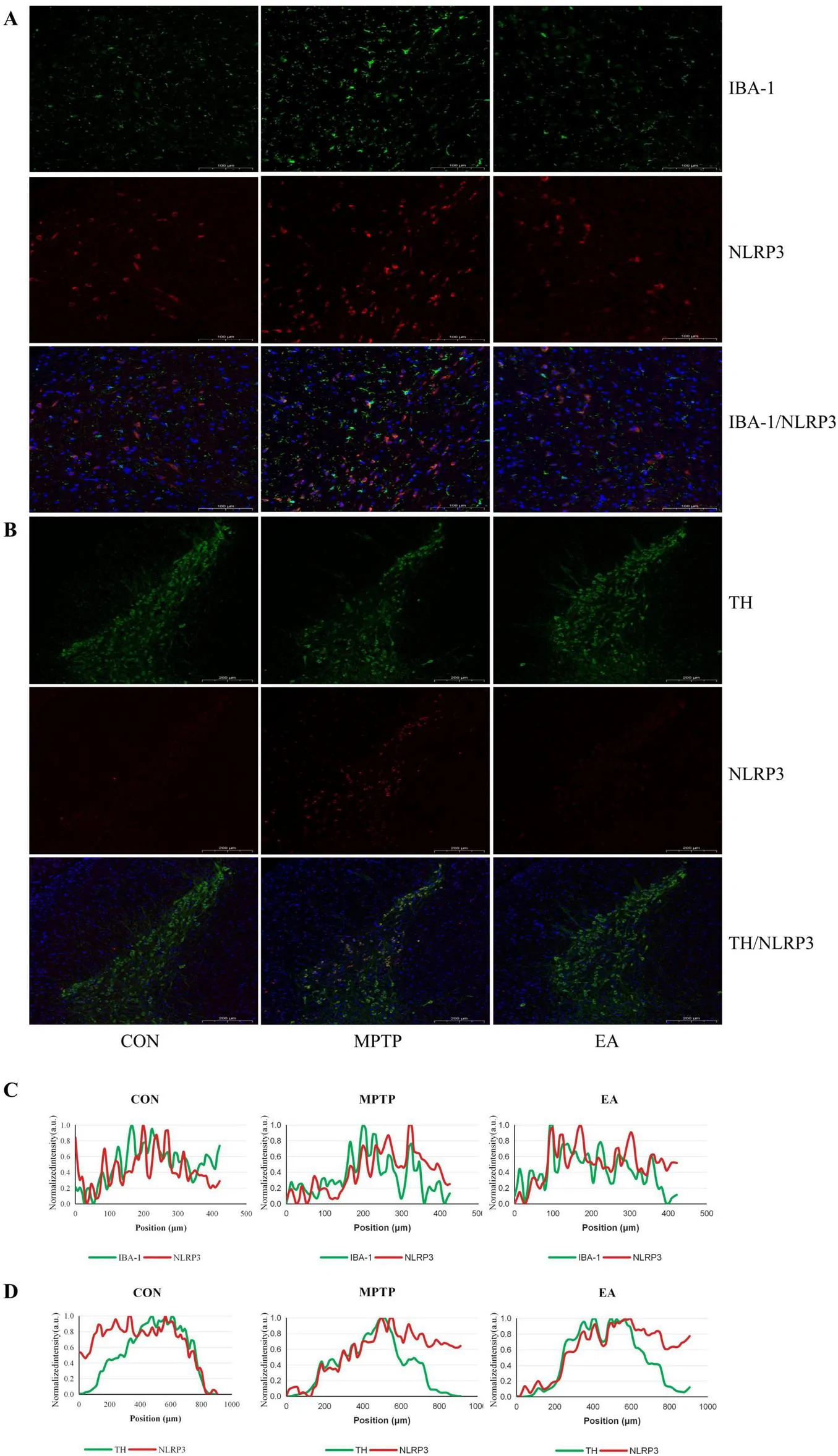
Electroacupuncture (EA) reduces NOD-, LRR- and pyrin domain-containing protein 3 (NLRP3) expression and its co-localization with dopaminergic neurons in the substantia nigra (SN) of MPTP mice. **(A)** Representative images showing IBA-1 (green) and NLRP3 (red) co-staining. Weak overlapping signals between IBA-1 and NLRP3 were observed. **(B)** Representative images showing tyrosine hydroxylase (TH) (green) and NLRP3 (red) co-staining. **(C)** Co-localization signal between IBA-1 and NLRP3. **(D)** Co-localization signal between TH and NLRP3. *n* = 5.

### Modulatory effect of EA on the pyroptosis pathway and TH expression in the SN of MPTP mice

Immunofluorescence analysis revealed significantly upregulated expression of ASC, Caspase-1, and GSDMD-N in the SN of MPTP mice compared to the control group (Figures 4A–D). This upregulation was notably suppressed following EA intervention (Figures 4A–D). To corroborate these findings and obtain a quantitative assessment of global protein changes in the SN, WB was performed. WB results confirmed a significant increase in NLRP3, ASC, Caspase-1, and GSDMD-N protein expression, alongside a significant decrease in TH expression, in the model group (Figures 5A,B and Supplementary Table 15). EA treatment significantly reversed these changes, reducing pyroptosis-related protein expression and restoring TH levels (Figures 5A,B and Supplementary Table 15). Quantitative analysis confirmed the changes observed in the representative immunohistochemical images, and the complete numerical results and statistical comparisons are provided in Supplementary Table 15.

**FIGURE 4:**
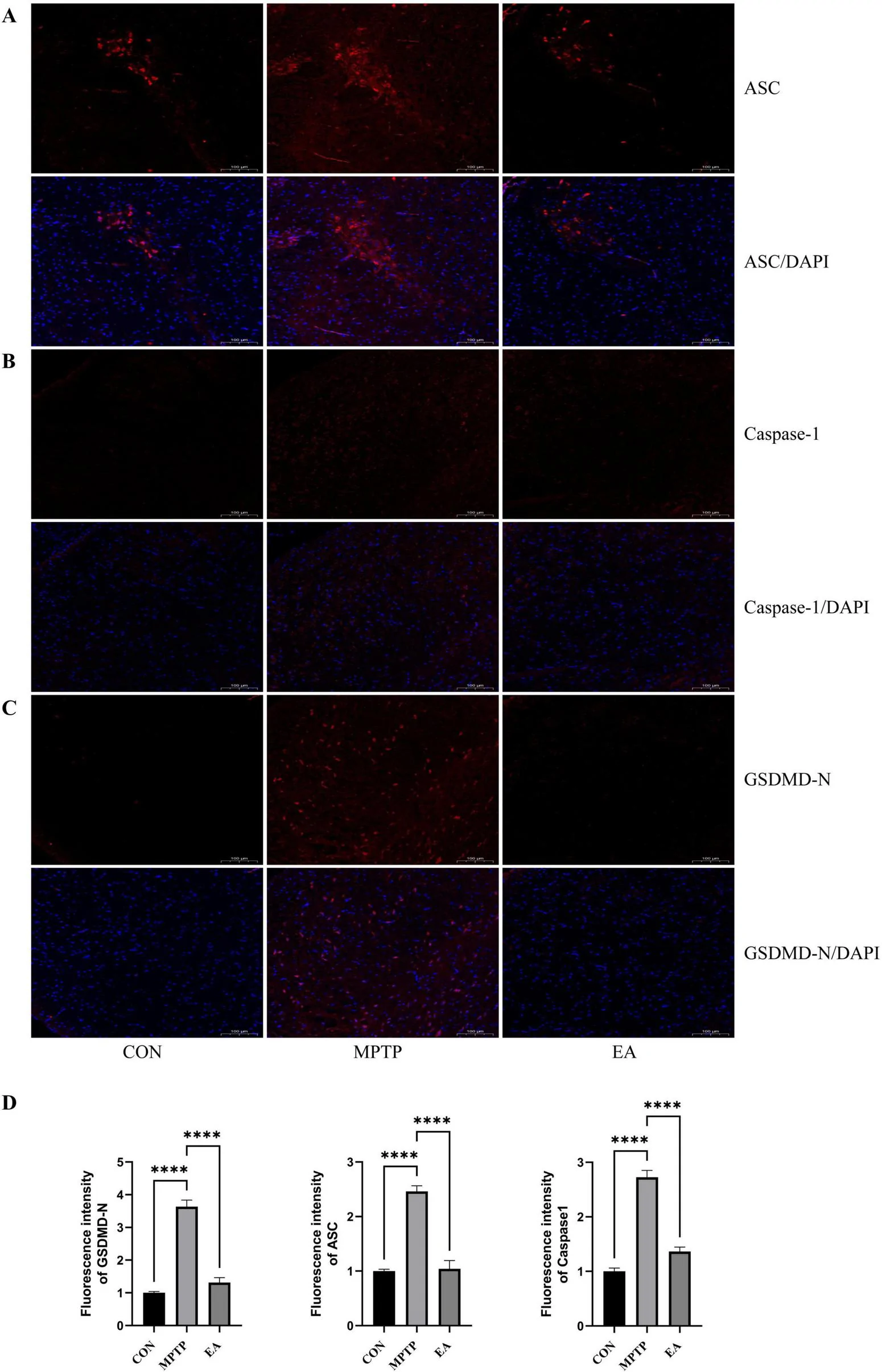
Electroacupuncture (EA) reduces ASC, Caspase1, and N-terminal fragment of gasdermin D (GSDMD-N) expression in the substantia nigra (SN) of MPTP mice. **(A)** Representative images showing ASC (red) and DAPI (blue). **(B)** Representative images showing Caspase-1 (red) and DAPI (blue). **(C)** Representative images showing GSDMD-N (red) and DAPI (blue). **(D)** Quantitative fluorescence intensity of ASC, Caspase-1 and GSDMD-N. Data are means ± SD. *n* = 5, ****P < 0.0001.

**FIGURE 5:**
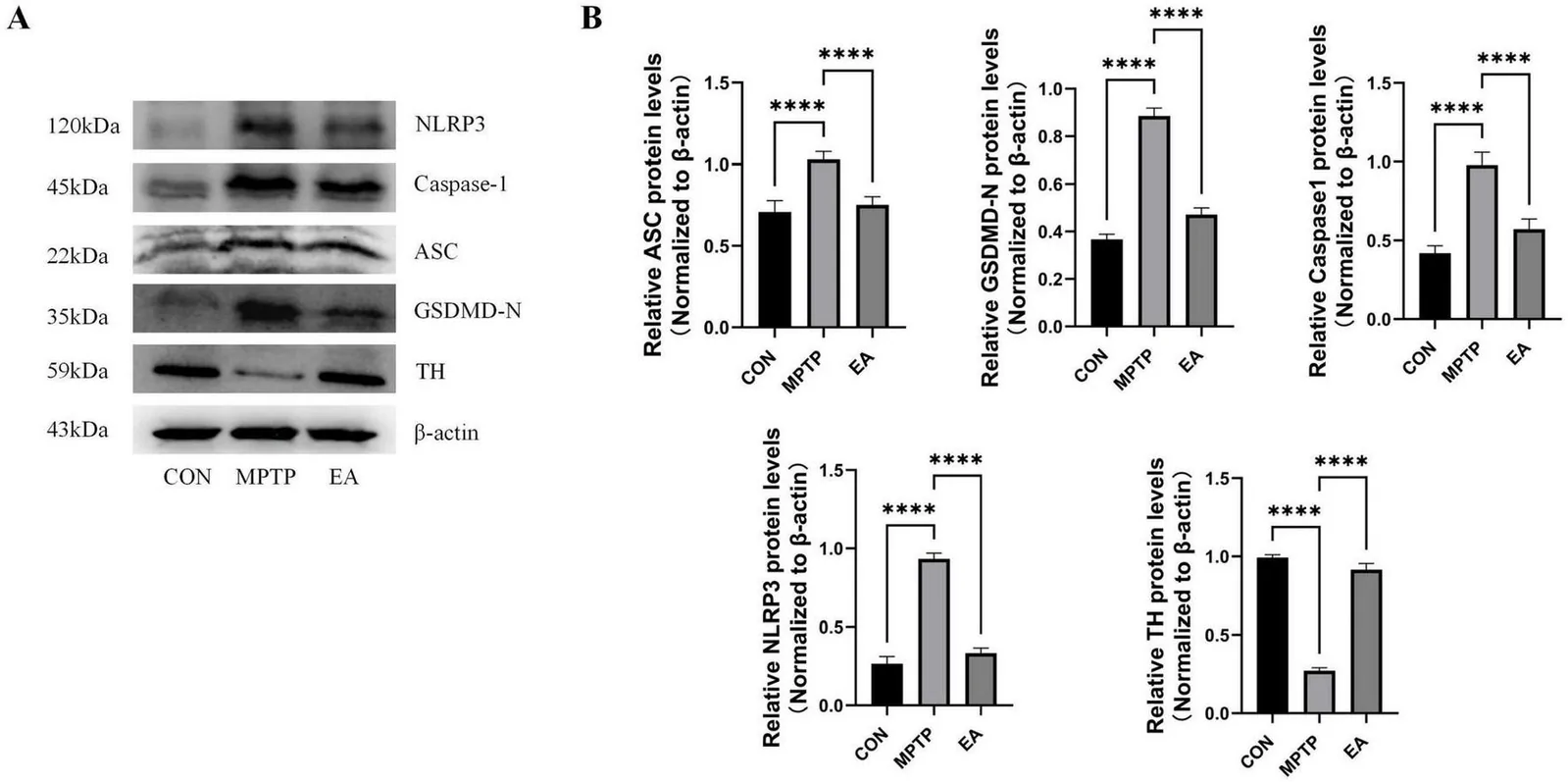
Electroacupuncture (EA) attenuates NOD-, LRR- and pyrin domain-containing protein 3 (NLRP3) inflammasome-mediated pyroptosis and restores tyrosine hydroxylase (TH) expression in the substantia nigra of MPTP mice. **(A)** Western blotting (WB) analysis of TH, NLRP3, Caspase1, N-terminal fragment of gasdermin D (GSDMD-N), ASC and β-actin. **(B)** Grayscale value calculation for TH, NLRP3, Caspase1, GSDMD-N and ASC. Data are means ± SD. *n* = 5, ****P < 0.0001.

### Behavioral alterations following EA and Nigericin administration in MPTP mice

In the open field test, compared with the control group, mice in the MPTP group exhibited significantly increased average locomotor speed and total movement distance (Figures 6A,B,D). EA intervention markedly ameliorated these behavioral abnormalities, as evidenced by significant reductions in both average locomotor speed and total distance traveled (Figures 6A,B,D). Compared with the MPTP group, Nigericin administration further exacerbated the hyperactive behavioral phenotype, resulting in increased average locomotor speed and total movement distance (Figures 6A,B,D). However, no significant differences were observed between the EA+Nigericin group and the MPTP+Nigericin group in terms of locomotor speed or total distance traveled within the central area (Figures 6A,B,D). Furthermore, the pole test demonstrated that, compared with the control group, MPTP mice required significantly more time to descend from the top to the bottom of the pole, whereas EA treatment significantly shortened the descent time (Figure 6C). Nigericin administration further prolonged the time required for MPTP mice to complete the pole descent task compared with the MPTP group (Figure 6C). However, EA treatment failed to significantly reduce the descent time in mice receiving combined MPTP and Nigericin administration (Figure 6C).

**FIGURE 6:**
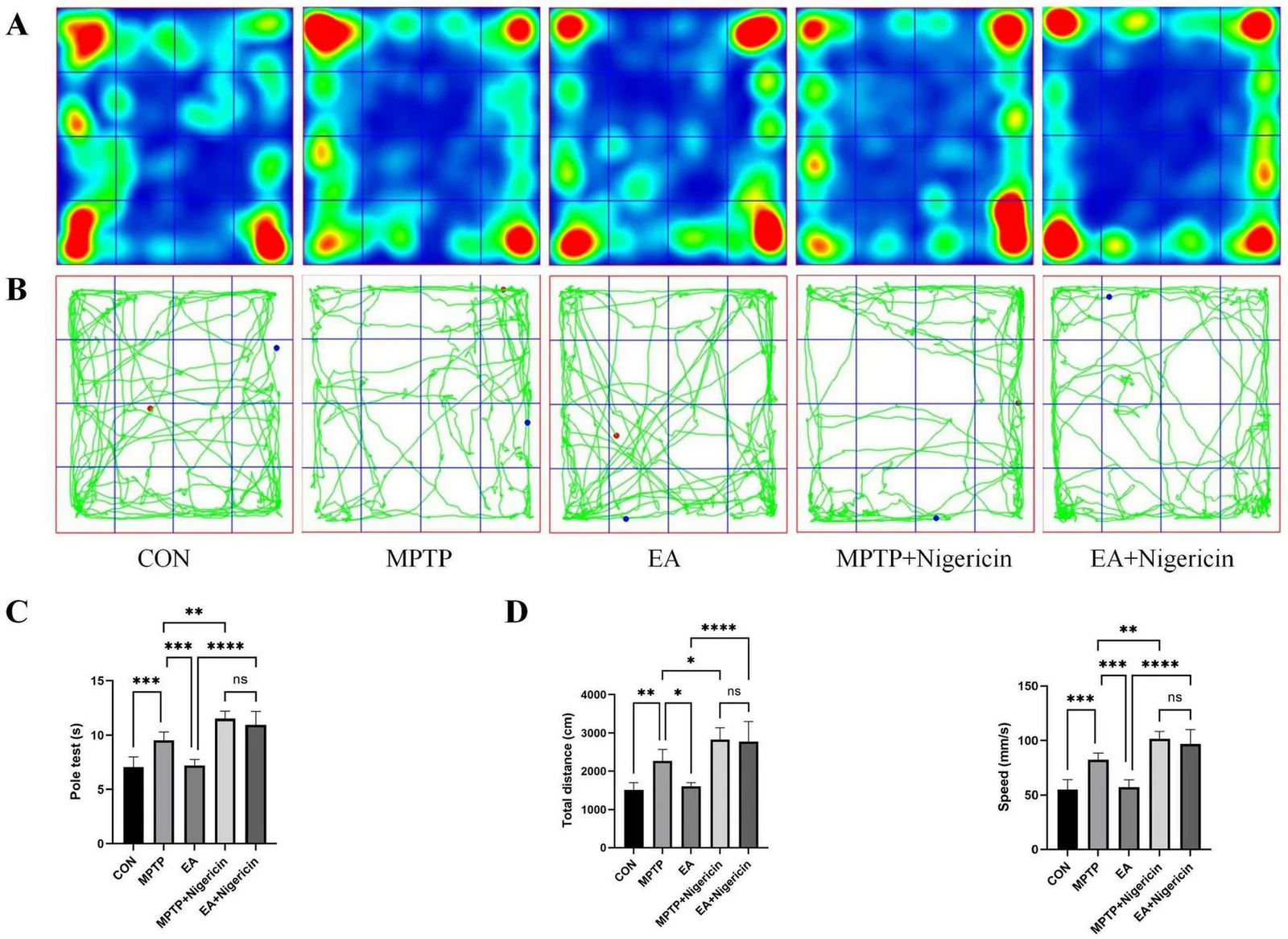
Nigericin-mediated activation of NOD-, LRR- and pyrin domain-containing protein 3 (NLRP3) inflammasome attenuates the beneficial effects of electroacupuncture (EA) on motor deficits in MPTP mice. **(A)** Heat map representation of the time of mice in the open field test. **(B)** Pathway maps of mice in the open field test. **(C)** Climbing down time in pole test. **(D)** Total distance and speed of mice in the open field test. Data are means ± SD. *n* = 5, *P < 0.05, **P < 0.01, ***P < 0.001, ****P < 0.0001, *nsP* > 0.05.

### Effects of EA and Nigericin administration on the SN and striatum of MPTP mice

Immunohistochemistry analysis revealed that, compared with the control group, MPTP administration significantly reduced the number of TH-positive dopaminergic neurons in the SN and decreased TH expression levels in the striatum (Figures 7A,B and Supplementary Tables 5, 6). EA intervention markedly reversed these alterations, as evidenced by increased dopaminergic neuron survival in the SN and enhanced TH expression in the striatum (Figures 7A,B and Supplementary Tables 5, 6). However, Nigericin administration further aggravated MPTP-induced dopaminergic neuronal loss in the SN and reduction of striatal TH expression. Under these conditions, subsequent EA treatment failed to produce significant improvements in either SN dopaminergic neuron numbers or striatal TH expression (Figures 7A,B and Supplementary Tables 5, 6). Consistent with the IHC findings, HE staining demonstrated that MPTP mice exhibited increased neuronal swelling and necrotic changes in the SN compared with control mice (Figure 7C and Supplementary Table 7). EA treatment significantly alleviated these pathological alterations, resulting in reduced neuronal necrosis (Figure 7C and Supplementary Table 7). In contrast, Nigericin administration further exacerbated MPTP-induced neuronal damage, and additional EA intervention did not significantly ameliorate neuronal necrosis in Nigericin-treated mice (Figure 7C and Supplementary Table 7). Furthermore, IF analysis revealed marked activation of microglia in the SN of MPTP mice, as indicated by increased microglial activation markers. EA treatment significantly suppressed MPTP-induced microglial activation. However, Nigericin administration further enhanced microglial activation induced by MPTP, and EA treatment failed to reverse this effect in Nigericin-treated mice (Figure 7D and Supplementary Table 8). Quantitative analysis confirmed the changes observed in the representative immunohistochemical images, and the complete numerical results and statistical comparisons are provided in Supplementary Tables 5–8.

**FIGURE 7:**
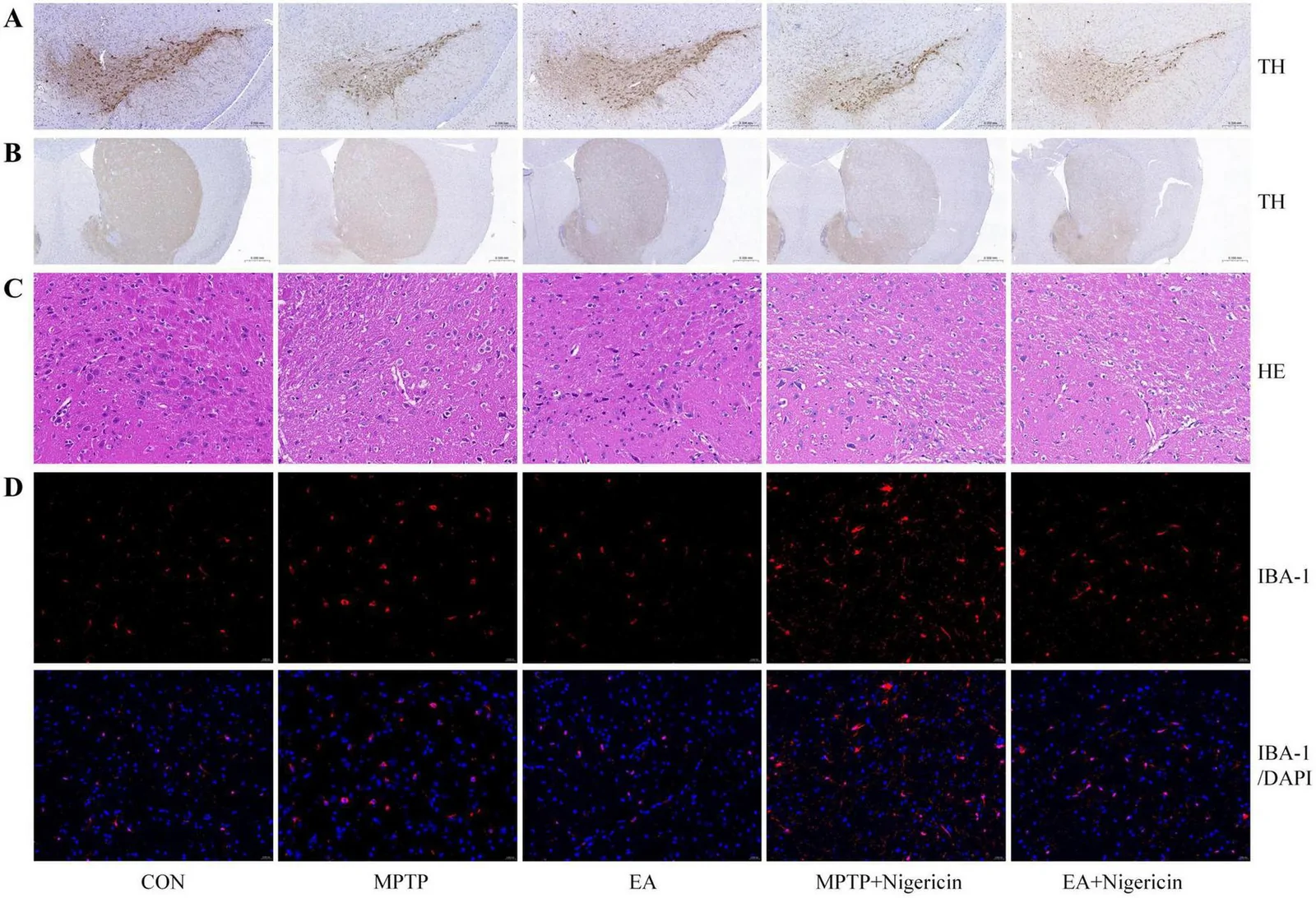
Effects of electroacupuncture (EA) and Nigericin administration on dopaminergic neuronal survival, neuronal injury, and microglial activation in the substantia nigra (SN) and striatum of MPTP mice. **(A)** Tyrosine hydroxylase (TH) immunohistochemistry (IHC) staining in the SN. **(B)** TH IHC staining in the striatum. **(C)** Hematoxylin and eosin (HE) staining showing necrotic changes in the SN. **(D)** IBA1-positive microglial activation in the SN. *n* = 5.

### Effects of EA and Nigericin administration on pyroptosis-related signaling pathways in the SN of MPTP mice

Immunofluorescence analysis revealed that, compared with the control group, MPTP administration significantly increased the expression levels of pyroptosis-related proteins, including NLRP3, ASC, Caspase-1, and GSDMD-N, in the SN (Figures 8A–D and Supplementary Table 9). EA intervention markedly suppressed the upregulation of these pyroptosis-associated proteins in the SN (Figures 8A–D and Supplementary Table 9). Furthermore, activation of NLRP3 inflammasome by Nigericin further enhanced the MPTP-induced upregulation of NLRP3, ASC, Caspase-1, and GSDMD-N expression. Under these conditions, EA treatment exhibited a mild inhibitory tendency toward pyroptosis-related protein expression; however, these effects did not reach statistical significance (Figures 8A–D and Supplementary Table 9). Given that IF and IHC analyses primarily reflect regional protein expression patterns within tissue sections, WB was subsequently performed to provide a comprehensive quantitative assessment of overall protein alterations in the SN. WB analysis confirmed that MPTP administration significantly increased the protein expression levels of NLRP3, ASC, Caspase-1, and GSDMD-N, accompanied by a marked reduction in TH expression, compared with the control group (Figures 9A,B). EA treatment significantly reversed these alterations by suppressing pyroptosis-related protein expression and restoring TH levels (Figures 9A,B). Similarly, under conditions of Nigericin-induced NLRP3 activation, EA treatment showed a slight inhibitory effect on the elevated expression of pyroptosis-associated proteins; however, the differences did not reach statistical significance. Quantitative analysis confirmed the changes observed in the representative immunohistochemical images, and the complete numerical results and statistical comparisons are provided in Supplementary Table 19.

**FIGURE 8:**
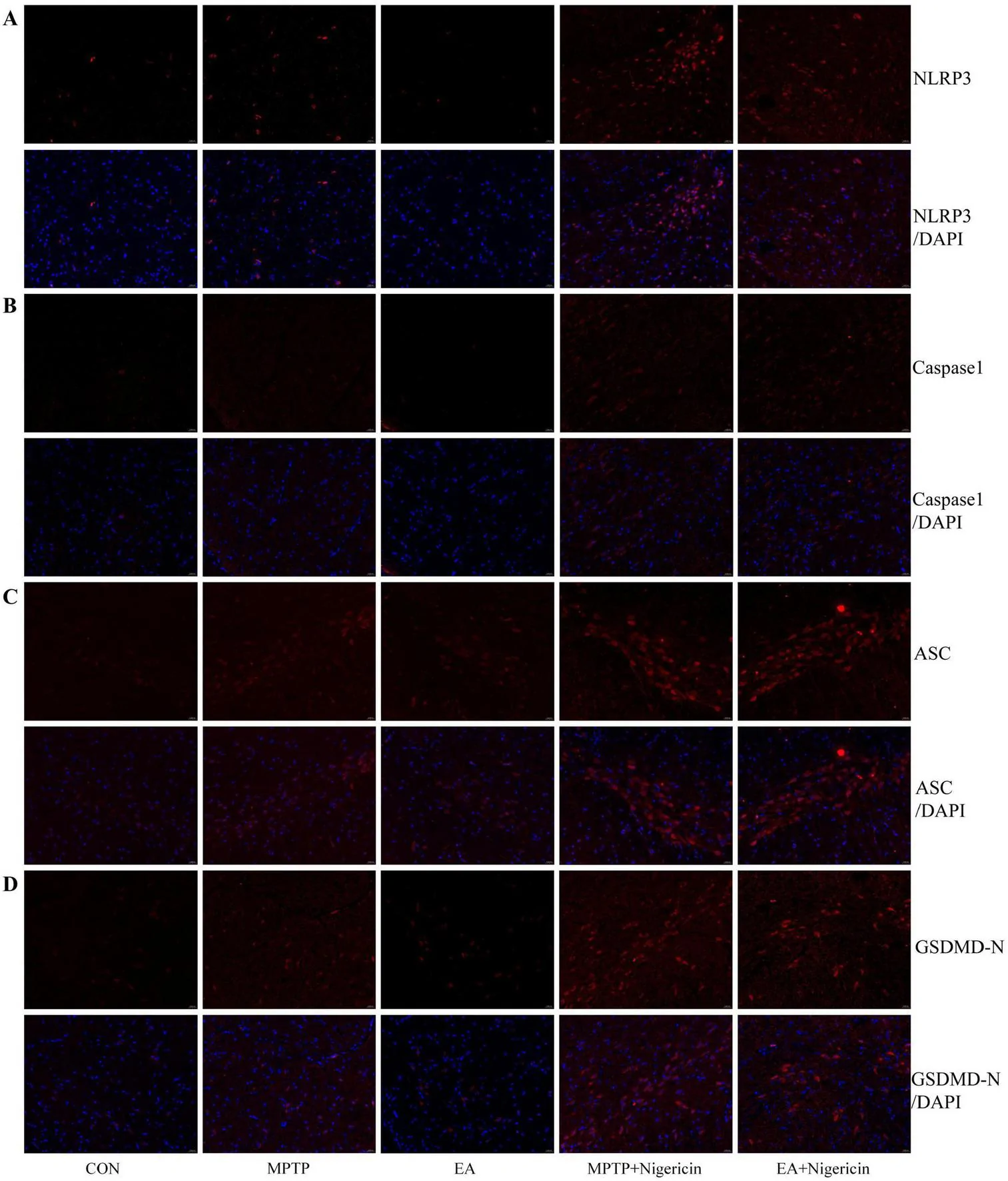
Nigericin activation of the NOD-, LRR- and pyrin domain-containing protein 3 (NLRP3) inflammasome counteracts the inhibitory effect of electroacupuncture (EA) on pyroptosis-related signaling in the substantia nigra (SN) of MPTP mice. **(A–D)** Representative IF images showing the expression of NLRP3, ASC, Caspase-1, and N-terminal fragment of gasdermin (GSDMD-N) in the SN. *n* = 5.

**FIGURE 9:**
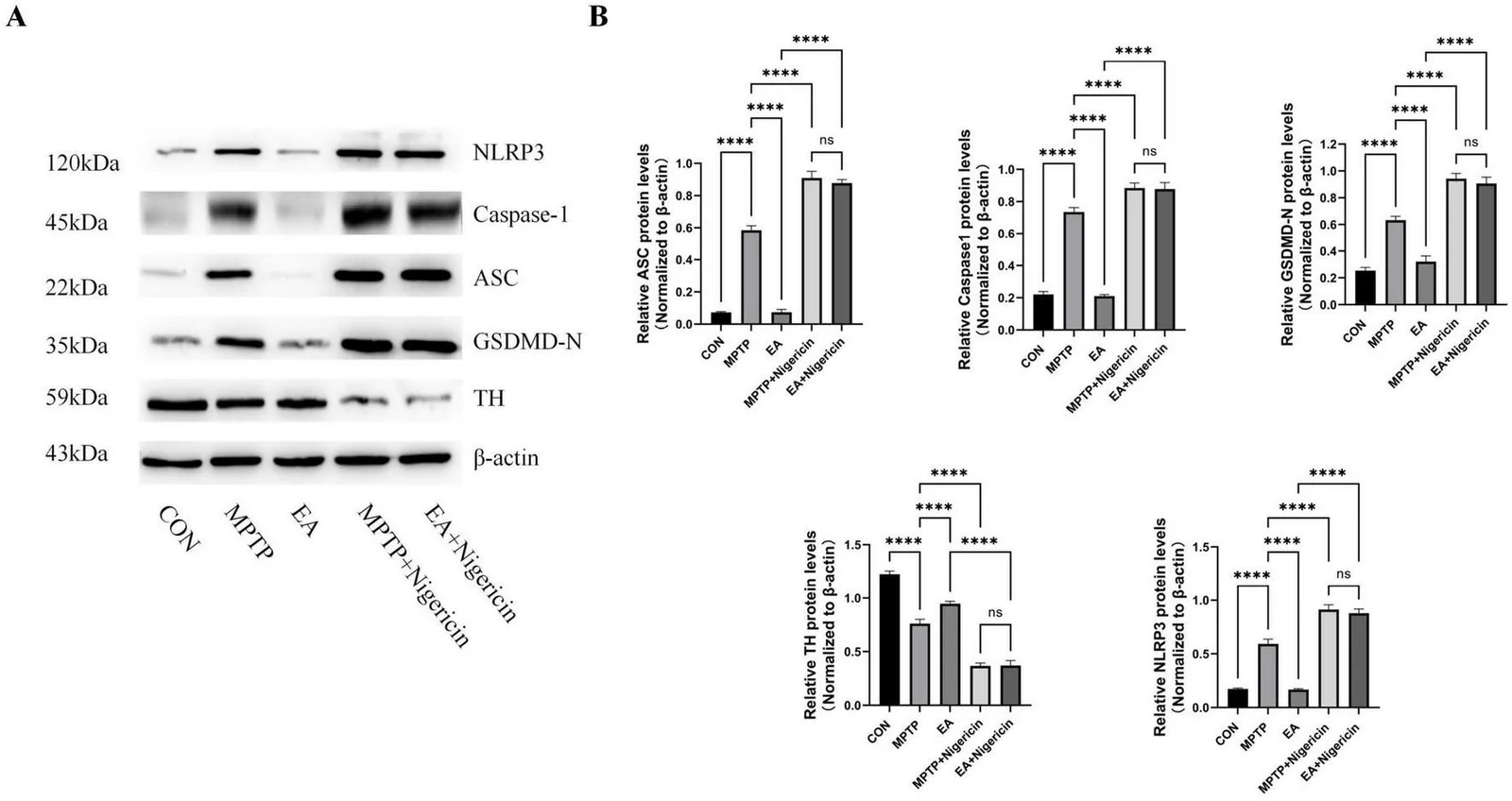
Nigericin activation of the NOD-, LRR- and pyrin domain-containing protein 3 (NLRP3) inflammasome counteracts the inhibitory effect of electroacupuncture (EA) on pyroptosis-related signaling in the substantia nigra (SN) of MPTP mice. **(A)** Representative Western blotting (WB) images showing the expression of tyrosine hydroxylase (TH), NLRP3, ASC, Caspase-1, N-terminal fragment of gasdermin (GSDMD-N) and β-actin in the SN. **(B)** Grayscale value calculation for TH, NLRP3, Caspase1, GSDMD-N and ASC. Data are means ± SD. *n* = 5, ****P < 0.0001, *nsP* > 0.05.

### Behavioral alterations following EA and MCC950 administration in MPTP mice

In the open field test, compared with the control group, mice in the MPTP group exhibited significantly increased average locomotor speed and total movement distance (Figures 10A,B,D). EA intervention markedly ameliorated these behavioral abnormalities, as evidenced by significant reductions in both average locomotor speed and total movement distance (Figures 10A,B,D). Compared with the MPTP group, MCC950 administration further reduced the average locomotor speed and total movement distance in MPTP-treated mice (Figures 10A,B,D). Notably, no significant differences were observed between the MPTP+MCC950 group and the EA group in terms of locomotor speed or total movement distance. Furthermore, additional EA treatment in MPTP+MCC950 mice failed to produce further improvements in these behavioral parameters (Figures 10A,B,D). Furthermore, the pole test demonstrated that, compared with the control group, MPTP mice required significantly more time to descend from the top to the bottom of the pole, whereas EA treatment significantly shortened the descent time (Figure 10C). Compared with the MPTP group, MCC950 administration further reduced the time required for MPTP mice to complete the pole descent task (Figure 10C). However, additional EA intervention in MPTP+MCC950 mice did not result in a significant reduction in descent time. Consistently, no significant difference was observed between the MPTP+MCC950 group and the EA group regarding pole descent time (Figure 10C).

**FIGURE 10:**
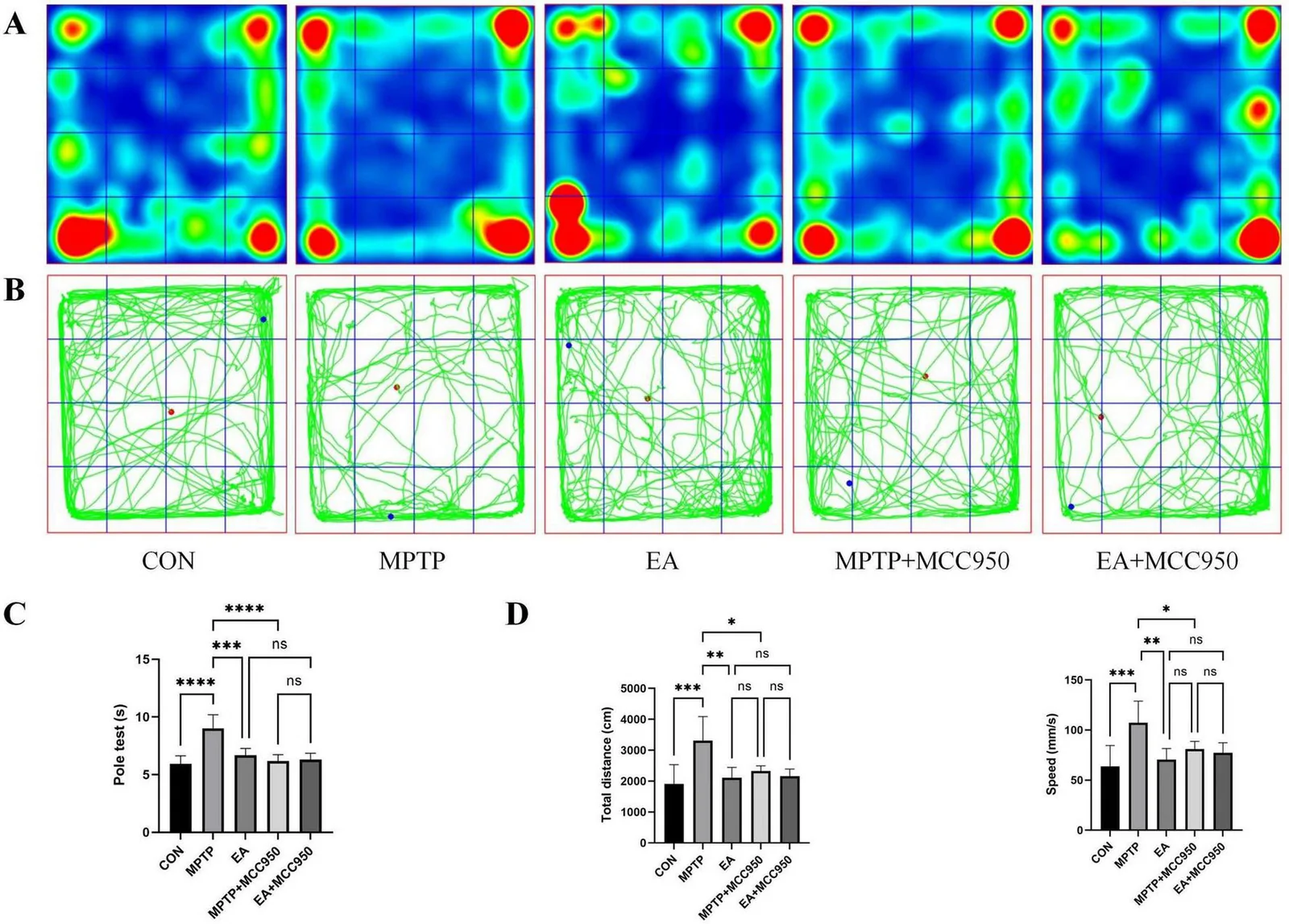
MCC950-mediated inhibition of NOD-, LRR- and pyrin domain-containing protein 3 (NLRP3) inflammasome activation mimics the protective effects of electroacupuncture (EA) on motor deficits in MPTP mice. **(A)** Heat map representation of the time of mice in the open field test. **(B)** Pathway maps of mice in the open field test. **(C)** Climbing down time in pole test. **(D)** Total distance and speed of mice in the open field test. Data are means ± SD. *n* = 5, *P < 0.05, **P < 0.01, ***P < 0.001, ****P < 0.0001, *nsP* > 0.05.

### Effects of EA and MCC950 administration on the SN and striatum of MPTP mice

Immunohistochemistry analysis revealed that, compared with the control group, MPTP administration significantly reduced the number of TH-positive dopaminergic neurons in the SN and decreased TH expression levels in the striatum (Figures 11A,B and Supplementary Tables 10, 11). EA intervention markedly reversed these alterations, as evidenced by increased survival of dopaminergic neurons in the SN and restored TH expression in the striatum (Figures 11A,B and Supplementary Tables 10, 11). Similarly, MCC950 treatment significantly attenuated MPTP-induced dopaminergic neuronal loss in the SN and reduction of striatal TH expression (Figures 11A,B and Supplementary Tables 10, 11). However, additional EA intervention following MCC950 administration failed to produce further improvements in either SN dopaminergic neuron numbers or striatal TH expression (Figures 11A,B and Supplementary Tables 10, 11). Consistent with the IHC findings, HE staining demonstrated that MPTP mice exhibited a significant increase in neuronal necrosis in the SN compared with control mice (Figure 11C and Supplementary Table 12). EA treatment effectively reduced the number of necrotic neurons (Figure 11C and Supplementary Table 12). MCC950 administration also significantly alleviated MPTP-induced neuronal necrosis in the SN (Figure 11C and Supplementary Table 12). However, under MCC950 treatment conditions, additional EA intervention did not result in further changes in the number of necrotic neurons (Figure 11C and Supplementary Table 12). Furthermore, IF analysis revealed marked microglial activation in the SN of MPTP mice, whereas EA treatment significantly suppressed this activation (Figure 11D and Supplementary Table 13). MCC950 administration attenuated MPTP-induced microglial activation, and additional EA intervention failed to provide further inhibitory effects under these conditions (Figure 11D and Supplementary Table 13). Consistently, no significant difference in microglial activation was observed between the MPTP+MCC950 group and the EA group (Figure 11D and Supplementary Table 13). Quantitative analysis confirmed the changes observed in the representative immunohistochemical images, and the complete numerical results and statistical comparisons are provided in Supplementary Table 13.

**FIGURE 11:**
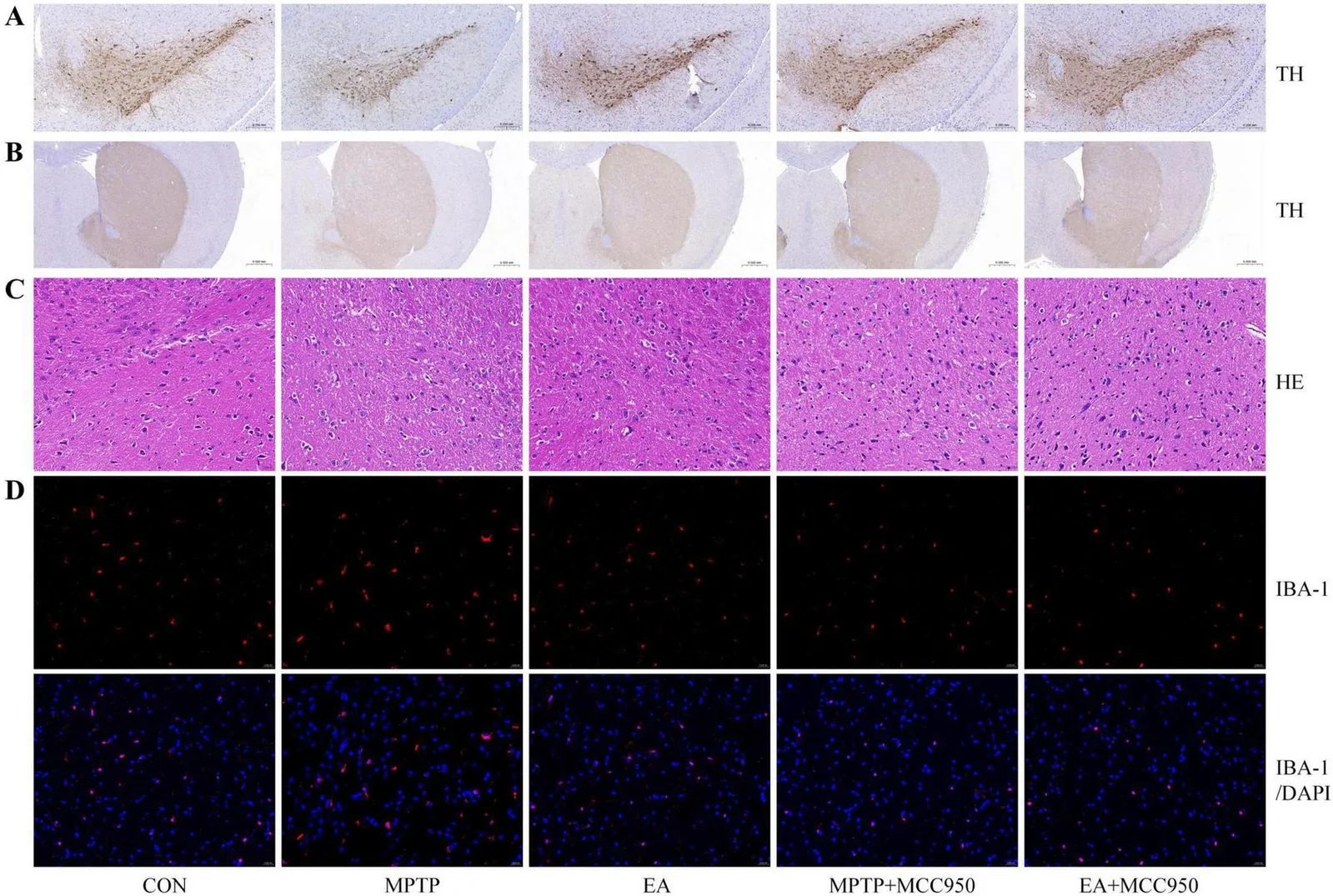
Effects of electroacupuncture (EA) and MCC950 administration on dopaminergic neuronal survival, neuronal injury, and microglial activation in the substantia nigra (SN) and striatum of MPTP mice. **(A)** Tyrosine hydroxylase (TH) immunohistochemistry (IHC) staining in the SN. **(B)** TH IHC staining in the striatum. **(C)** Hematoxylin and eosin (HE) staining showing necrotic changes in the SN. **(D)** IBA1-positive microglial activation in the SN. *n* = 5.

### Effects of EA and MCC950 administration on pyroptosis-related signaling pathways in the SN of MPTP mice

Immunofluorescence and IHC analyses revealed that, compared with the control group, MPTP administration significantly increased the expression levels of pyroptosis-related proteins, including ASC, Caspase-1, and GSDMD-N, in the SN (Figures 12A–D and Supplementary Table 14). EA intervention markedly suppressed the upregulation of these pyroptosis-associated proteins in the SN. Furthermore, MCC950 treatment significantly attenuated the MPTP-induced upregulation of NLRP3, ASC, Caspase-1, and GSDMD-N expression. Under these conditions, additional EA treatment failed to produce further significant improvements in the expression levels of pyroptosis-related proteins (Figures 12A–D and Supplementary Table 14). Given that IF and IHC analyses primarily reflect regional protein expression patterns within tissue sections, WB was subsequently performed to provide a comprehensive quantitative assessment of overall protein alterations in the SN. WB analysis confirmed that, compared with the control group, MPTP administration significantly increased the protein expression levels of NLRP3, ASC, Caspase-1, and GSDMD-N, accompanied by a marked reduction in TH expression (Figures 13A,B). EA treatment significantly reversed these alterations by suppressing pyroptosis-related protein expression and restoring TH levels (Figures 13A,B). Similarly, under conditions of MCC950-mediated inhibition of MPTP-induced pyroptosis-related protein upregulation, additional EA treatment did not result in further significant improvements. Moreover, no significant differences in the expression levels of NLRP3, ASC, Caspase-1, and GSDMD-N were observed between the EA group and the MPTP+MCC950 group (Figures 13A,B). Quantitative analysis confirmed the changes observed in the representative immunohistochemical images, and the complete numerical results and statistical comparisons are provided in Supplementary Table 14.

**FIGURE 12:**
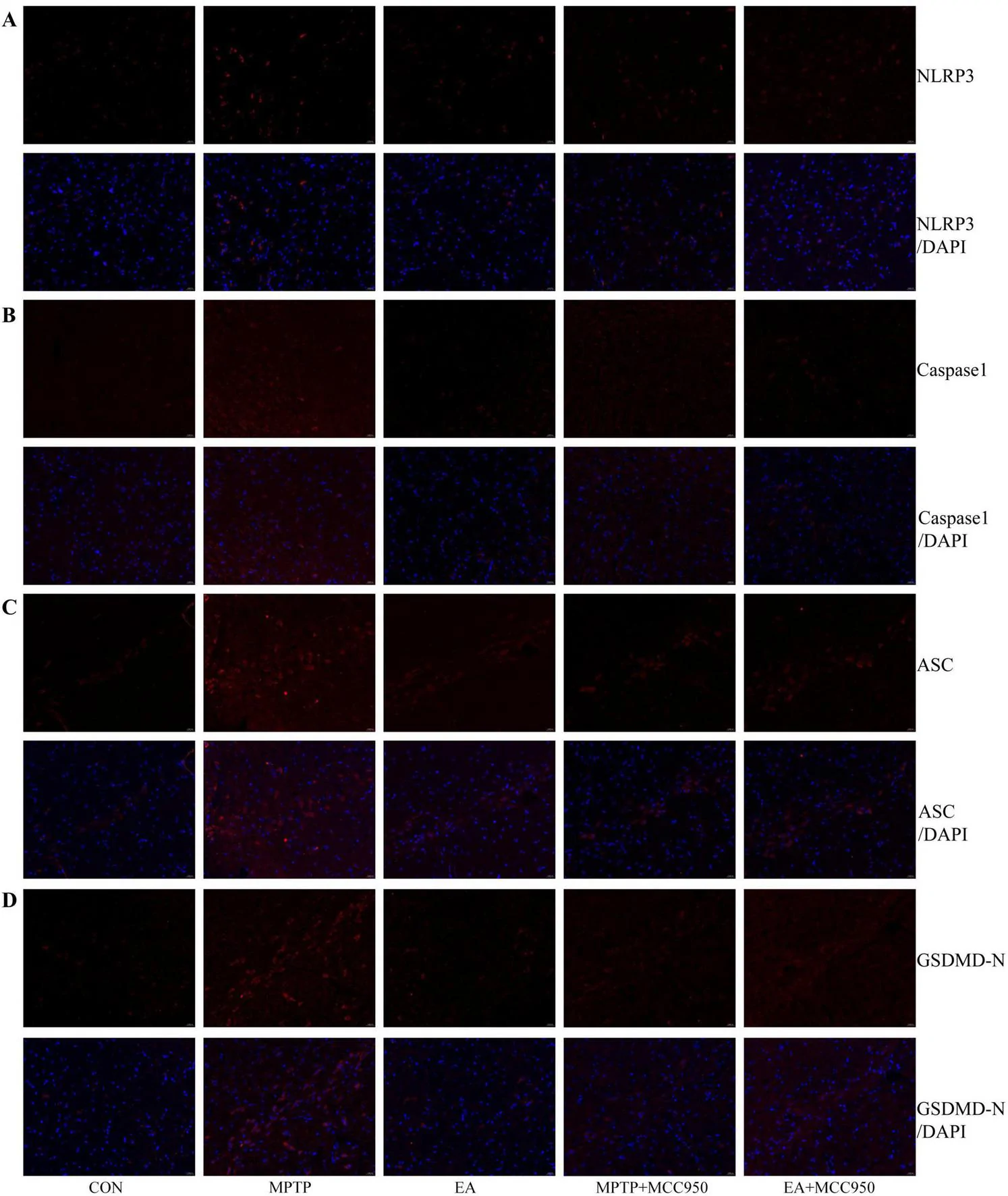
MCC950-mediated inhibition of the NOD-, LRR- and pyrin domain-containing protein 3 (NLRP3) inflammasome mimics the inhibitory effect of electroacupuncture (EA) on pyroptosis-related signaling in the substantia nigra (SN) of MPTP mice. (A–D) Representative IF images showing the expression of NLRP3, ASC, Caspase-1, and N-terminal fragment of gasdermin (GSDMD-N) in the SN. *n* = 5.

**FIGURE 13:**
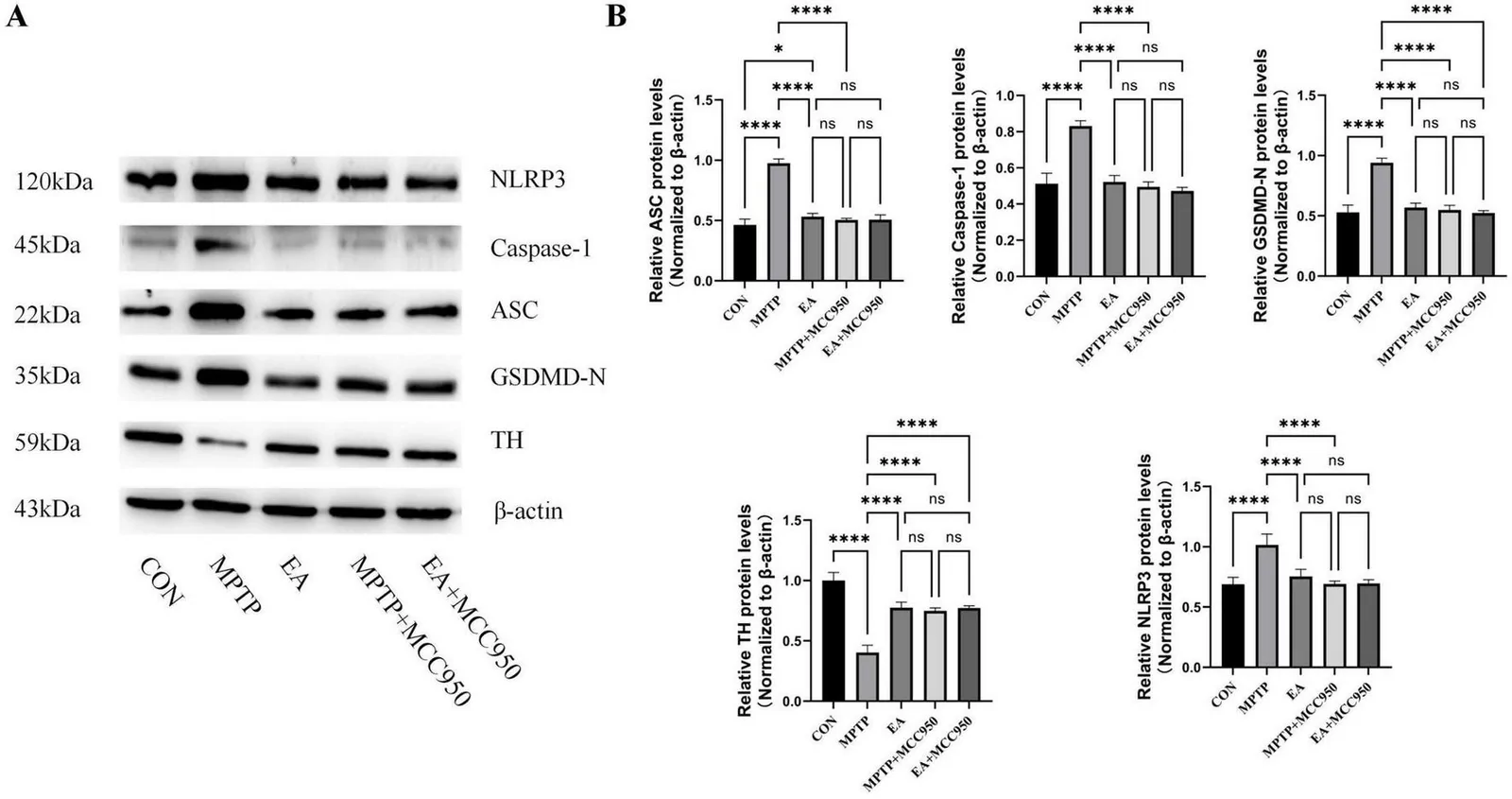
MCC950-mediated inhibition of the NOD-, LRR- and pyrin domain-containing protein 3 (NLRP3) inflammasome mimics the inhibitory effect of electroacupuncture (EA) on pyroptosis-related signaling in the substantia nigra (SN) of MPTP mice. **(A)** Representative Western blotting (WB) images showing the expression of tyrosine hydroxylase (TH), NLRP3, ASC, Caspase-1, N-terminal fragment of gasdermin (GSDMD-N), and β-actin in the SN. **(B)** Grayscale value calculation for TH, NLRP3, Caspase-1, GSDMD-N, and ASC. Data are presented as mean ± SD. *n* = 5, *P < 0.05, ****P < 0.0001, ns*P* > 0.05.

## Discussion

Parkinson’s disease is a common neurodegenerative disorder characterized primarily by motor symptoms, including bradykinesia, resting tremor, rigidity, and postural instability, and is frequently accompanied by non-motor symptoms such as depression and sleep disturbances. Clinical studies have demonstrated that EA stimulation can ameliorate both motor and non-motor symptoms in patients with PD. However, the underlying mechanisms by which EA improves motor dysfunction in PD remain largely unclear. In the present study, we provide evidence that EA alleviates motor deficits and neuroinflammation in a PD mouse model. The following findings support our conclusions: (1) EA treatment improved motor dysfunction in MPTP mice; (2) EA treatment inhibited the activation of the NLRP3 inflammasome; and (3) the therapeutic effects of EA were mediated, at least partially, through the suppression of NLRP3 inflammasome-mediated pyroptosis.

1-methyl-4-phenyl-1,2,3,6-tetrahydropyridine (MPTP) (30 mg/ kg, intraperitoneally) was administered daily for 5 consecutive days to establish a PD mouse model, as previously described in multiple studies (Sun et al., 2018; Zhu et al., 2019). Previous studies have demonstrated that 100 Hz EA stimulation at GV20 and GV14 produces superior therapeutic effects compared with stimulation at 50, 2, or 0 Hz (Jia et al., 2010). Therefore, 100 Hz EA stimulation at GV20 and GV14 was selected in the present study. According to previous reports, MPTP mice exhibit motor dysfunction, reduced dopamine and serotonin levels in the striatum, degeneration of dopaminergic neurons in the SN, and decreased TH expression (Sun et al., 2018; Zhu et al., 2019).

In the present study, MPTP-treated mice exhibited prolonged pole-descent time, which was shortened by EA, whereas locomotor speed and total movement distance in the open field test were unexpectedly increased by MPTP and subsequently reduced by EA. Similar open-field hyperactivity after MPTP exposure has been reported previously, including increases in movement time, locomotor speed, and total distance that were reversed by EA (Wang et al., 2013). This apparent discrepancy may partly relate to the relatively high MPTP dose used in our subacute model (30 mg/kg, intraperitoneally) (Jackson-Lewis and Przedborski, 2007), because high-dose MPTP exposure may affect the serotonergic system and contribute to hyperlocomotor phenotypes (Chia et al., 1996, 1999). Acute stress associated with transfer to the testing environment may also increase dopamine release in the striatum, nucleus accumbens, and medial prefrontal cortex (Abercrombie Keefe et al., 1989), potentially inducing transient compensatory locomotor activation in animals with nigrostriatal injury (Zigmond and Stricker, 1989). Importantly, the open field and pole tests evaluate different aspects of motor function. The open field test primarily measures spontaneous horizontal activity, including anxiety-related thigmotaxis or wall-following behavior, which may manifest as purposeless “pseudo-hyperactivity” (Palasz et al., 2017). In contrast, the pole test assesses goal-directed motor coordination and postural control, requiring vertical orientation, turning, gripping, and regulation of the center of gravity through coordinated integration of the basal ganglia, cerebellum, sensory systems, and motor cortex (Airaksinen and Saarma, 2002). Prolonged pole-descent time is therefore considered a characteristic indicator of bradykinesia and postural instability in PD models (Zhao et al., 2008). Thus, the increased open-field activity and impaired pole-test performance are not necessarily contradictory but may reflect task-dependent involvement of distinct motor circuits, with compensatory or stress-related spontaneous hyperactivity occurring alongside impaired nigrostriatal-dependent complex motor execution.

Subsequently, we performed double IF staining for Iba-1 and TH to determine the cellular localization of pyroptotic signaling. The results demonstrated that the majority of IBA-1-positive and NLRP3-positive cells exhibited minimal overlap, suggesting that pyroptosis was unlikely to primarily occur in microglia in our model. Subsequent double immunofluorescence staining for TH and NLRP3 further confirmed that NLRP3 expression was predominantly localized within dopaminergic neurons. Furthermore, triple immunofluorescence staining for NLRP3, IBA-1, and TH performed in independent batches of PD mice further validated that pyroptosis predominantly occurred in dopaminergic neurons rather than microglia. After identifying the cellular population undergoing pyroptosis, we further examined the assembly of the NLRP3 inflammasome and its downstream pyroptotic effectors. Our findings demonstrated that EA treatment effectively reversed the MPTP-induced upregulation of Caspase-1, ASC, and GSDMD-N expression in the SN, indicating that EA suppresses NLRP3 inflammasome-mediated pyroptotic signaling in dopaminergic neurons. Previous studies have predominantly emphasized microglial NLRP3 inflammasome activation in PD models (Gordon et al., 2018; Huang et al., 2024; Lee et al., 2019). In contrast, NLRP3 immunoreactivity in the present study showed greater overlap with TH-positive cells than with IBA-1-positive microglia. This difference may reflect variations in the experimental model, MPTP administration regimen, observation time, disease stage, cellular response, or image-analysis method. Nevertheless, the present findings do not exclude the contribution of microglial inflammasome signaling to MPTP-induced neuroinflammation and dopaminergic neuronal injury.

To further evaluate the necessity of NLRP3 activation in the therapeutic effects of EA, PD mice were treated with the NLRP3 inflammasome activator Nigericin. Nigericin, a K^+^/H^+^ ionophore, induces rapid intracellular K^+^ depletion by promoting K^+^ efflux and H^+^ influx, and the subsequent reduction in intracellular K^+^ concentration is considered a critical event in NLRP3 inflammasome activation (Rivers-Auty and Brough, 2015; Shavit and San Pietro, 1967). To date, no studies have reported the application of Nigericin in subacute PD models. Therefore, in the present study, the dosage regimen was determined based on protocols previously used in other disease models, followed by preliminary experiments. Our results demonstrated that administration of Nigericin at 2 mg/kg aggravated motor deficits in MPTP mice while significantly reducing mortality. Therefore, this dosage was selected for subsequent intraperitoneal administration. In the open field test, EA treatment effectively reversed the typical behavioral alterations induced by MPTP, including reduced center-area exploration and prolonged peripheral zone occupancy. Notably, compared with mice receiving MPTP alone, mice treated with MPTP combined with Nigericin exhibited significantly increased total movement distance and average locomotor speed. As discussed above, high-dose MPTP exposure may influence serotonergic signaling and induce stress-mediated striatal dopamine release, thereby contributing to partial behavioral activation. However, the further enhancement of locomotor activity following Nigericin administration on the basis of MPTP treatment suggests that additional pathophysiological mechanisms may be involved. As a potent activator of the NLRP3 inflammasome, Nigericin markedly exacerbates pyroptotic responses in the SN of MPTP-treated mice, leading to excessive release of pro-inflammatory cytokines, including IL-1β and IL-18. These inflammatory mediators may contribute to abnormal locomotor behaviors through multiple mechanisms. First, direct disruption of motor circuits by neuroinflammation: cytokines such as IL-1β and IL-18 may directly affect neuronal activity in the thalamus or cortex, reducing neuronal excitation thresholds and diminishing sensitivity to inhibitory signals transmitted from the basal ganglia. This may result in abnormal spontaneous neuronal firing and increased purposeless, stereotypic, or agitation-like motor activity (Foster et al., 2021; Kim et al., 2017). Second, disruption of neurotransmitter system homeostasis: inflammatory microenvironments can alter the release, reuptake, and receptor function of critical neurotransmitters, including glutamate and γ-aminobutyric acid (GABA), thereby transiently disturbing the balance between excitation and inhibition within the basal ganglia–thalamocortical circuitry. For example, excessive glutamatergic excitation may mimic abnormal activation of the direct pathway and subsequently promote involuntary motor activity (Kakegawa et al., 2011; Xu et al., 2025). Third, dysregulation of ascending modulatory systems: as mentioned above, high-dose MPTP may affect serotonergic signaling originating from the raphe nuclei. Systemic and central inflammation induced by Nigericin may further disrupt the regulatory functions of ascending modulatory systems, including the raphe nuclei and locus coeruleus, impairing their feedback control over basal ganglia and cortical circuits. This may lead to increased overall motor excitability and reduced behavioral inhibition, manifesting as anxiety-like, exploratory, or stereotypic hyperactivity (Benarroch, 2009; Hopwood and Stamford, 2001; Judge et al., 2006). Collectively, these findings suggest that superimposed acute neuroinflammation on an existing PD pathological background can induce complex and non-linear alterations in motor behaviors. This observation provides potential experimental evidence for atypical symptoms such as restlessness and agitation observed in certain stages of PD, suggesting that neuroinflammation may represent an important contributor to these behavioral manifestations. In MPTP mice receiving combined Nigericin and EA treatment, the behavioral improvements mediated by EA were largely abolished, consistent with the findings observed in the pole test. Similarly, pyroptosis-related molecular alterations exhibited comparable trends with behavioral changes. Based on these findings, the beneficial effects of EA on motor phenotypes and its inhibitory effects on pyroptosis-associated signaling were significantly attenuated following Nigericin-mediated NLRP3 activation, suggesting that the neuroprotective effects of EA are primarily mediated, at least partially, through suppression of NLRP3 inflammasome-mediated pyroptotic pathways.

Considering that previous studies have demonstrated the involvement of EA in regulating oxidative stress and apoptosis in PD models, we further treated PD mice with the NLRP3 inhibitor MCC950 to investigate the role of NLRP3 signaling in EA-mediated neuroprotection. Our results showed that MCC950 suppressed the MPTP-induced upregulation of NLRP3, ASC, GSDMD-N, and Caspase-1 expression. However, additional EA treatment did not produce further reductions in these pyroptosis-related markers. The comparable effects observed between EA and MCC950 may be attributed to their convergence on common upstream regulatory mechanisms. MCC950 directly targets the NLRP3 protein itself, whereas extensive evidence indicates that the neuroprotective effects of EA involve multi-target regulation, including modulation of upstream signals responsible for NLRP3 inflammasome activation. For example, EA can regulate Toll-like receptor-related signaling pathways and inhibit NF-κB nuclear translocation, thereby reducing the transcription of NLRP3 and pro-IL-1β at the upstream level (Quiroz-González et al., 2025). In addition, EA has been shown to improve mitochondrial dysfunction and reduce the release of mitochondrial reactive oxygen species and damaged mitochondrial DNA, which serve as critical endogenous danger signals involved in NLRP3 inflammasome activation (Zhong et al., 2016). Therefore, MCC950 may directly interfere with the activation conformation of the NLRP3 protein, whereas EA may suppress NLRP3 activation indirectly by restoring mitochondrial homeostasis, reducing oxidative stress, and inhibiting microglial activation, thereby eliminating upstream danger signals that trigger NLRP3 inflammasome assembly. Although acting at different regulatory levels, both interventions ultimately disrupt NLRP3 inflammasome assembly and reduce downstream pyroptotic effector activation, resulting in highly comparable suppression of pyroptosis-related signaling and the absence of significant differences between treatment groups. This mechanism may also explain why combined EA and MCC950 treatment failed to produce additional synergistic effects, even when NLRP3 inflammasome activation was pharmacologically inhibited. The pharmacological findings further support the involvement of NLRP3-related signaling in the effects of EA. Nigericin aggravated nigrostriatal injury and weakened the protective effects of EA, whereas MCC950 produced effects comparable to those of EA. The absence of an obvious additive effect between EA and MCC950 suggests that their neuroprotective actions may involve partially overlapping NLRP3-related mechanisms. This interpretation is consistent with previous studies showing that pharmacological inhibition of NLRP3 attenuates dopaminergic neurodegeneration in MPTP- and α-synuclein-based PD models (Gordon et al., 2018; Lee et al., 2019). However, the lack of an additive effect does not establish NLRP3 as a direct molecular target of EA, nor does it demonstrate that EA and MCC950 act through completely identical mechanisms.

In conclusion, this study demonstrates that EA treatment may ameliorate motor dysfunction in a mouse model of PD by suppressing NLRP3 inflammasome-mediated pyroptosis. Mechanistically, the neuroprotective effects of EA appear to be closely associated with the inhibition of NLRP3 inflammasome activation. However, several limitations should be acknowledged. First, this study primarily focused on NLRP3-mediated pyroptotic signaling and did not investigate the potential contributions of other cell death pathways, such as oxidative stress-induced damage and apoptosis, to PD pathogenesis. Although the use of NLRP3 agonists and inhibitors provided evidence that pyroptosis may represent a predominant pathological process during the early stage of disease, multiple cellular mechanisms are likely to interact and contribute to disease progression at later stages. Second, the observation period in the present study was relatively short (only 2 weeks), which may not fully capture the dynamic pathological changes occurring throughout the progression of PD. Third, the behavioral analyses were conducted using five animals per group. Although consistent changes were observed in the open-field and pole tests and were supported by the histological, molecular, and pharmacological findings, the small sample size limits the statistical precision and generalizability of the behavioral results. These findings should therefore be interpreted cautiously and require confirmation in independent, prospectively powered animal cohorts. Fourth, only male mice were included in the present study. Previous studies have reported sex-dependent behavioral and neurochemical responses to MPTP, although these differences may vary according to the toxin dose, administration regimen, genetic background, and experimental endpoint (Alam et al., 2016; Antzoulatos et al., 2010). Therefore, the present findings cannot be directly generalized to female mice, and it remains unknown whether the effects of EA on NLRP3 inflammasome-related signaling are sex dependent. Future studies should include both sexes and employ an appropriately powered factorial design to evaluate sex-by-MPTP and sex-by-EA interactions. Despite these limitations, the present findings extend previous studies implicating NLRP3-associated inflammatory signaling and pyroptosis in the effects of EA in experimental PD. The combined behavioral, histological, molecular, and pharmacological results suggest that the neuroprotective effects of EA in MPTP-treated mice are associated with attenuation of NLRP3 inflammasome activation and downstream GSDMD-dependent pyroptosis-related signaling in the substantia nigra. The localization of NLRP3 immunoreactivity in TH-positive cells further suggests the potential involvement of dopaminergic neurons; however, direct cell-specific execution of pyroptosis remains to be established. Thus, these findings provide additional mechanistic support for further investigation of EA in PD rather than definitive evidence of a dopaminergic neuron-specific mechanism. Future studies should employ longer treatment and follow-up periods, as well as chronic or progressive PD models, to evaluate the durability of EA-mediated neuroprotection and its regulation of interactions among multiple pathological pathways. Further investigations are also required to identify the critical upstream molecular targets through which EA modulates NLRP3 inflammasome activation.

## Data Availability

The raw data supporting the conclusions of this article will be made available by the authors, without undue reservation.
