# Supplementary material for: Electroacupuncture inhibits pyroptosis to improve motor function in an MPTP-induced mouse model of Parkinson’s disease
Source: Front Aging Neurosci. 2026 Sep 30;18:1968826. doi: 10.3389/fnagi.2026.1968826 (PMC13661978; doi:10.3389/fnagi.2026.1968826)
Supplement: Supplementary file 1 [file Data_Sheet_1.docx]

**Histological and immunofluorescence image acquisition and quantification**

Coronal brain sections containing the substantia nigra (approximately −2.92 to −3.64 mm relative to bregma) were identified according to the Paxinos and Franklin mouse brain atlas. The substantia nigra pars compacta was delineated bilaterally using atlas-based anatomical landmarks, including its location lateral to the ventral tegmental area, dorsal to the substantia nigra pars reticulata, and medial to the cerebral peduncle. Three anatomically matched sections separated by approximately 120 μm were analyzed per mouse, and three non-overlapping fields covering the medial, central, and lateral portions of the substantia nigra pars compacta were acquired from each hemisphere of each section.

Images for the same marker were acquired using identical microscope, objective, resolution, exposure time or laser power, detector gain, and offset settings. Acquisition parameters were established using control sections to avoid signal saturation and were maintained unchanged across all groups within the same staining batch. Image analysis was performed using ImageJ software (National Institutes of Health, Bethesda, MD, USA) by an investigator blinded to group allocation. Background fluorescence measured in an adjacent region without specific staining was subtracted from the signal within the predefined region of interest. When threshold-based segmentation was required, the same threshold was applied to all images within the corresponding staining batch.

A TH-positive cell profile was defined as a clearly immunoreactive neuronal soma containing an identifiable nucleus. TH-positive cell profiles were counted bilaterally within the predefined substantia nigra pars compacta region. IBA-1, NLRP3, ASC, total Caspase-1, and GSDMD-N immunofluorescence was quantified as background-corrected mean fluorescence intensity within the same region of interest. For hematoxylin–eosin staining, neurons displaying shrunken cell bodies, cytoplasmic eosinophilia or vacuolation, and pyknotic or hyperchromatic nuclei were classified as morphologically injured. Neuronal injury was expressed as the percentage of injured neuronal profiles relative to the total number of neuronal profiles in the analyzed fields.

Values obtained from all fields and sections of the same mouse were averaged to generate one animal-level value. Thus, the mouse, rather than an individual section or microscopic field, was used as the statistical unit (n = 5 mice per group). Relative fluorescence measurements were normalized to the mean value of the control group, which was defined as 100%.
