# Supplementary material for: Electroacupuncture inhibits pyroptosis to improve motor function in an MPTP-induced mouse model of Parkinson’s disease
Source: Front Aging Neurosci. 2026 Sep 30;18:1968826. doi: 10.3389/fnagi.2026.1968826 (PMC13661978; doi:10.3389/fnagi.2026.1968826)
Supplement: Supplementary file 2 [file Data_Sheet_2.docx]

Table S1 Number of dopaminergic neurons in SN（ ±s）

| Group | n | Dopaminergic neurons (n) |
| --- | --- | --- |
| CON | 5 | 139.8±14.89 |
| MPTP | 5 | 97.0±12.94* |
| EA | 5 | 114.2±8.93^#^ |

Compared with the CON, **P* < 0.05；compared with the MPTP, ^#^*P* < 0.05; SN, substantia nigra

Table S2 TH protein expression levels in striatum（ ±s）

| Group | | n | TH protein expression level |
| --- | --- | --- | --- |
| CON | | 5 | 1.00±0.20 |
|  | MPTP | 5 | 0.35±0.11* |
| EA | | 5 | 0.69±0.17^#^ |

Compared with the CON, **P* < 0.05；compared with the MPTP, ^#^*P* < 0.05

Table S3 Number of necrotic neurons in SN（±s）

| Group | n | Necrotic neurons (n) |
| --- | --- | --- |
| CON | 5 | 6.80±2.86 |
| MPTP | 5 | 16.40±3.43* |
| EA | 5 | 10.80±2.86^#^ |

Compared with the CON, **P* < 0.05；compared with the MPTP, ^#^*P* < 0.05; SN, substantia nigra

Table S4 Relative fluorescence intensity of IBA-1 in SN（±s）

| Group | n | Relative fluorescence intensity of IBA-1 |
| --- | --- | --- |
| CON | 5 | 1.00±0.17 |
| MPTP | 5 | 2.62±0.23* |
| EA | 5 | 1.10±0.31^#^ |

Compared with the CON, **P* < 0.05；compared with the MPTP, ^#^*P* < 0.05; SN, substantia nigra

Table S5 Number of dopaminergic neurons in SN（±s）

| Group | n | Dopaminergic neurons (n) |
| --- | --- | --- |
| CON | 5 | 154.60±11.19 |
| MPTP | 5 | 107.20±6.10* |
| EA | 5 | 127.00±7.58^#^ |
| MPTP +Nigericin | 5 | 89.40±6.11^&^ |
| EA +Nigericin | 5 | 92.60±11.85^λ^ |

Compared with the CON, **P*<0.05; compared with the MPTP, ^#^*P*<0.05, ^&^*P*<0.05；compared with the EA, ^λ^*P*<0.05; SN, substantia nigra

Table S6 TH protein expression levels in striatum（±s）

| Group | n | TH protein expression level |
| --- | --- | --- |
| CON | 5 | 0.23 ±0.03 |
| MPTP | 5 | 0.10±0.02* |
| EA | 5 | 0.16±0.02^#^ |
| MPTP +Nigericin | 5 | 0.05±0.02^&^ |
| EA +Nigericin | 5 | 0.06±0.01^λ^ |

Compared with the CON, **P*<0.05；compared with the MPTP, ^#^*P*<0.05，^&^*P*<0.05；compared with the EA, ^λ^*P*<0.05

Table S7 Number of necrotic neurons in SN（±s）

| Group | n | Necrotic neurons (n) |
| --- | --- | --- |
| CON | 5 | 7.20±2.68 |
| MPTP | 5 | 19.60±3.65* |
| EA | 5 | 12.00±4.00^#^ |
| MPTP +Nigericin | 5 | 29.00±4.53^&^ |
| EA +Nigericin | 5 | 28.80±5.63^λ^ |

Compared with the CON, **P*<0.05; compared with the MPTP, ^#^*P*<0.05, ^&^*P*<0.05；compared with the EA, ^λ^*P*<0.05; SN, substantia nigra

Table S8 Relative fluorescence intensity of IBA-1 in SN（±s）

| Group | n | Relative fluorescence intensity of IBA-1 |
| --- | --- | --- |
| CON | 5 | 1.00±0.07 |
| MPTP | 5 | 2.71±0.03* |
| EA | 5 | 1.29±0.03^#^ |
| MPTP +Nigericin | 5 | 3.63±0.03^&^ |
| EA +Nigericin | 5 | 3.46±0.03^λ^ |

Compared with the CON, **P*<0.05; compared with the MPTP, ^#^*P*<0.05, ^&^*P*<0.05；compared with the EA, ^λ^*P*<0.05; SN, substantia nigra

Table S9 Relative fluorescence intensity of pyroptosis‑related indicators in SN（±s）

| Group | n | NLRP3 | Caspase-1 | ASC | GSDMD-N |
| --- | --- | --- | --- | --- | --- |
| CON | 5 | 1.00±0.01 | 1.00±0.01 | 1.00±0.01 | 1.00±0.02 |
| MPTP | 5 | 1.89±0.01* | 1.77±0.01* | 1.40±0.02* | 2.23±0.02* |
| EA | 5 | 1.03±0.01^#^ | 1.32±0.00^#^ | 1.10±0.01^#^ | 1.52±0.04^#^ |
| MPTP +Nigericin | 5 | 2.65±0.03^&^ | 2.43±0.02^&^ | 2.10±0.04^&^ | 3.66±0.03^&^ |
| EA +Nigericin | 5 | 2.57±0.02^λ^ | 2.32±0.02^λ^ | 2.02±0.02^λ^ | 3.61±0.04^λ^ |

Compared with the CON, **P*<0.05; compared with the MPTP, ^#^*P*<0.05, ^&^*P*<0.05；compared with the EA, ^λ^*P*<0.05; SN, substantia nigra

Table S10 Number of dopaminergic neurons in SN（±s）

| Group | n | Dopaminergic neurons (n) |
| --- | --- | --- |
| CON | 5 | 151.6±9.81 |
| MPTP | 5 | 105.4±10.14* |
| EA | 5 | 126.4±6.54^#^ |
| MPTP +MCC950 | 5 | 124.6±9.84^&§^ |
| MPTP +MCC950 | 5 | 126.8±9.07^λ^ |

Compared with the CON, **P*<0.05; compared with the MPTP, ^#^*P*<0.05, ^&^*P*<0.05；compared with the EA，^λ^*P* > 0.05，^§^*P* > 0.05; SN, substantia nigra

Table S11 TH protein expression levels in striatum（±s）

| Group | n | TH protein expression level |
| --- | --- | --- |
| CON | 5 | 0.230 ± 0.038 |
| MPTP | 5 | 0.113 ± 0.032* |
| EA | 5 | 0.181 ± 0.017^#^ |
| MPTP +MCC950 | 5 | 0.189 ± 0.024^&§^ |
| MPTP +MCC950 | 5 | 0.193 ± 0.019^λ^ |

Compared with the CON, **P*<0.05; compared with the MPTP, ^#^*P*<0.05, ^&^*P*<0.05；compared with the EA，^λ^*P* > 0.05，^§^*P* > 0.05

Table S12 Number of necrotic neurons in SN（±s）

| Group | N | Necrotic neurons (n) |
| --- | --- | --- |
| CON | 5 | 8.0±3.16 |
| MPTP | 5 | 22.8±4.60* |
| EA | 5 | 12.6±2.30^#^ |
| MPTP +MCC950 | 5 | 12.2±4.21^&§^ |
| MPTP +MCC950 | 5 | 12.2±2.59^λ^ |

Compared with the CON, **P*<0.05; compared with the MPTP, ^#^*P*<0.05, ^&^*P*<0.05；compared with the EA，^λ^*P* > 0.05，^§^*P* > 0.05; SN, substantia nigra

Table S13 Relative fluorescence intensity of IBA-1 in SN（±s）

| Group | N | Relative fluorescence intensity of IBA-1 |
| --- | --- | --- |
| CON | 5 | 1.00±0.07 |
| MPTP | 5 | 2.50±0.06* |
| EA | 5 | 1.35±0.02^#^ |
| MPTP +MCC950 | 5 | 1.28±0.02^&§^ |
| MPTP +MCC950 | 5 | 1.32±0.03^λ^ |

Compared with the CON, **P*<0.05; compared with the MPTP, ^#^*P*<0.05, ^&^*P*<0.05；compared with the EA，^λ^*P* > 0.05，^§^*P* > 0.05; SN, substantia nigra

Table S14 Relative fluorescence intensity of pyroptosis‑related indicators in SN（±s）

| Group | n | NLRP3 | Caspase-1 | ASC | GSDMD-N |
| --- | --- | --- | --- | --- | --- |
| CON | 5 | 1.00 ± 0.01 | 1.00±0.01 | 1.00±0.01 | 1.00±0.02 |
| MPTP | 5 | 2.83 ± 0.02* | 2.21 ± 0.02* | 2.14 ± 0.04* | 1.91 ± 0.08* |
| EA | 5 | 1.40 ± 0.03^#^ | 1.38 ± 0.01^#^ | 1.31 ± 0.01^#^ | 1.25 ± 0.01^#^ |
| MPTP +MCC950 | 5 | 1.57 ± 0.03^&§^ | 1.45 ± 0.05^&§^ | 1.32 ± 0.01^&§^ | 1.28 ± 0.01^&§^ |
| MPTP +MCC950 | 5 | 1.48 ± 0.01^λ^ | 1.40 ± 0.04^λ^ | 1.36 ± 0.02^λ^ | 1.24 ± 0.03^λ^ |

Compared with the CON, **P*<0.05; compared with the MPTP, ^#^*P*<0.05, ^&^*P*<0.05；compared with the EA，^λ^*P* > 0.05，^§^*P* > 0.05; SN, substantia nigra

Table S15 Quantitative colocalization of NLRP3 with IBA-1 or TH in representative SN fields（±s）

| Colocalization pair | n | Colocalization metric | CON | MPTP | EA |
| --- | --- | --- | --- | --- | --- |
| IBA-1/NLRP3 | 5 | Pearson’s correlation coefficient (r) | 0.22±0.02 | 0.26±0.06* | 0.24±0.06^#^ |
| IBA-1/NLRP3 | 5 | Manders’ M1 coefficient (%) | 2.63 ± 1.65 | 13.25 ± 9.76* | 11.81 ± 12.95^#^ |
| TH/NLRP3 | 5 | Pearson’s correlation coefficient (r) | 0.61 ± 0.19 | 0.47 ± 0.18* | 0.55 ± 0.11^#^ |
| TH/NLRP3 | 5 | Manders’ M1 coefficient (%) | 66.81 ± 10.51 | 56.17 ± 9.21* | 63.41 ± 15.43^#^ |

Compared with the CON, **P*<0.05; compared with the MPTP, ^#^*P*<0.05; SN, substantia nigra
